# Comprehensive single-cell genome analysis at nucleotide resolution using the PTA Analysis Toolbox

**DOI:** 10.1016/j.xgen.2023.100389

**Published:** 2023-08-23

**Authors:** Sjors Middelkamp, Freek Manders, Flavia Peci, Markus J. van Roosmalen, Diego Montiel González, Eline J.M. Bertrums, Inge van der Werf, Lucca L.M. Derks, Niels M. Groenen, Mark Verheul, Laurianne Trabut, Cayetano Pleguezuelos-Manzano, Arianne M. Brandsma, Evangelia Antoniou, Dirk Reinhardt, Marc Bierings, Mirjam E. Belderbos, Ruben van Boxtel

**Affiliations:** 1 Princess Máxima Center for Pediatric Oncology, Utrecht, the Netherlands; 2 Oncode Institute, Utrecht, the Netherlands; 3 Department of Pediatric Oncology, Erasmus Medical Center – Sophia Children’s Hospital, Rotterdam, the Netherlands; 4 Hubrecht Institute, Royal Netherlands Academy of Arts and Sciences (KNAW) and UMC Utrecht, Utrecht, the Netherlands; 5 Department of Pediatric Hematology and Oncology, University Hospital Essen, Essen, Germany

**Keywords:** single-cell sequencing, whole-genome sequencing, primary template-directed amplification, whole-genome amplification, somatic mutations, mutational signatures, cancer, Fanconi anemia, hematopoietic stem cells, structural variants

## Abstract

Detection of somatic mutations in single cells has been severely hampered by technical limitations of whole-genome amplification. Novel technologies including primary template-directed amplification (PTA) significantly improved the accuracy of single-cell whole-genome sequencing (WGS) but still generate hundreds of artifacts per amplification reaction. We developed a comprehensive bioinformatic workflow, called the PTA Analysis Toolbox (PTATO), to accurately detect single base substitutions, insertions-deletions (indels), and structural variants in PTA-based WGS data. PTATO includes a machine learning approach and filtering based on recurrence to distinguish PTA artifacts from true mutations with high sensitivity (up to 90%), outperforming existing bioinformatic approaches. Using PTATO, we demonstrate that hematopoietic stem cells of patients with Fanconi anemia, which cannot be analyzed using regular WGS, have normal somatic single base substitution burdens but increased numbers of deletions. Our results show that PTATO enables studying somatic mutagenesis in the genomes of single cells with unprecedented sensitivity and accuracy.

## Highlights

• PTATO enables accurate detection of somatic mutations in single-cell WGS data
• Single base substitution artifacts caused by PTA are removed using machine learning
• PTATO includes indel and structural variant filtering of PTA-based WGS data
• An increase in deletions is detected in blood stem cells of Fanconi anemia patients

## Graphical abstract

**Figure:**
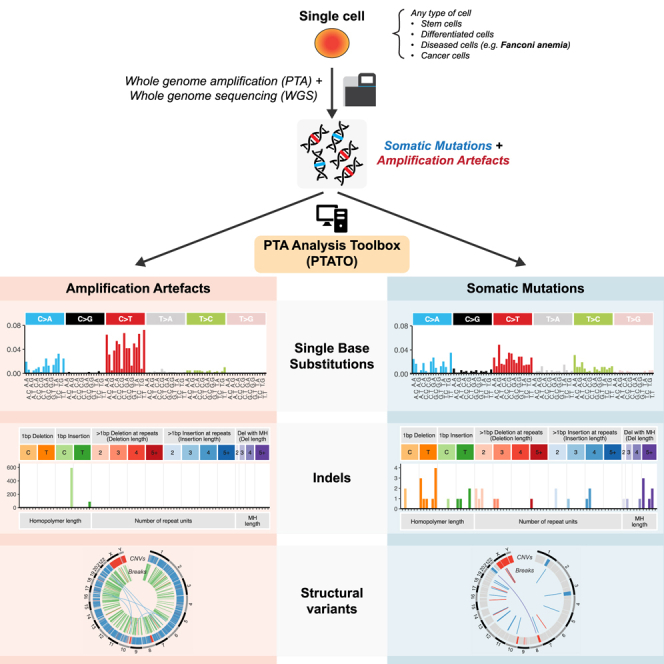

## Teaser

Detection of somatic mutations in single cells is challenging, in part because whole-genome amplification causes many artificial mutations. Middelkamp et al. developed the computational PTA Analysis Toolbox (PTATO) that can effectively filter artifacts from PTA-based WGS data, enabling accurate analyses of somatic mutations in single cells at nucleotide resolution.

## Introduction

Somatic mutations gradually accumulate in each cell during life, which can contribute to the development of age-related diseases, such as cancer.^1^^,^^2^^,^^3^ Due to the stochastic nature of mutation accumulation, each cell contains a unique set of somatic variants. Amplification of the genome of a single cell is required to obtain sufficient DNA for WGS. One approach for this is to catalog mutations in clonal structures that exist in tissues *in vivo*^4^ or after clonally expanding single cells isolated from tissues *in vitro.*^5^^,^^6^ However, these approaches can only be applied to cells that have the capacity to clonally expand such as stem cells, precluding analyses of many diseased and/or post-mitotic differentiated cell types.^7^ Examples of these are hematopoietic stem and progenitor cells (HSPCs) of patients with Fanconi anemia (FA), who suffer from progressive bone marrow failure and are predisposed to cancer due to an inherited deficiency of DNA repair.^8^^,^^9^^,^^10^ Much of the research into the mutagenic processes in FA HSPCs has been performed using mouse models,^11^^,^^12^^,^^13^ because primary HSPCs of human patients with FA are difficult to culture and clonally expand *in vitro.*^14^^,^^15^

An alternative method to clonal expansion is the use of whole-genome amplification (WGA) techniques to directly amplify DNA of single cells in enzymatic reactions. However, single-cell WGA technologies have traditionally been hindered by technical limitations due to uneven and erroneous amplification of the genome, leading to artificial mutations, noise in copy number profiles, and missing mutations due to allelic dropout.^16^ Recently, a novel WGA method, called primary template-directed amplification (PTA), was developed, which contains several critical improvements over the traditionally used multiple displacement amplification protocol.^17^ Although the amplification biases and allelic dropout rates of PTA are remarkably low, it still generates hundreds to thousands of false-positive single base substitutions and indels in each amplification reaction.^17^^,^^18^ Bioinformatic approaches, such as linked read analysis (LiRA)^19^ and SCAN2,^18^ have been developed to filter and analyze WGS data of WGA samples. However, these tools still have low detection sensitivities (∼10%–40%), and therefore most true variants are missed.^18^^,^^19^ Additionally, while PTA has the potential to enable structural variant (SV) detection in single cells, current tools are not optimized for PTA-based single-cell WGS data.

Here, we developed the PTA Analysis Toolbox (PTATO), which uses a machine learning model to accurately filter artifacts from PTA-based WGS data and is optimized for SV detection. We demonstrate the applicability of PTATO by analyzing the genomes of normal HSPCs of FA patients and show that, similar to current FA mouse models, these cells have an increased somatic deletion burden.

## Results

### Training a random forest model to filter PTA artifacts

**Figure 1:**
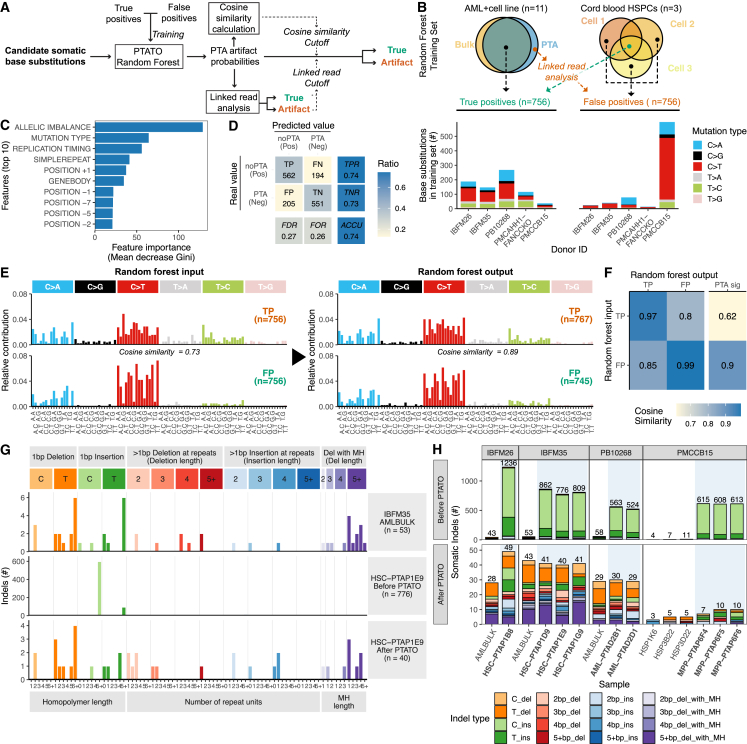
Accurate filtering of PTA artifacts using machine learning and recurrence filtering (A) Outline of the PTATO workflow to classify candidate base substitutions as true variants or PTA artifacts. The trained PTATO RF model calculates the probability that each variant is a PTA artifact. Subsequently it uses a linked read analysis and cosine similarity calculations to determine a sample-specific probability cutoff. (B) Overview of the samples and base substitutions that are used as PTA artifacts or true variants to train the RF model. (C) Importance of the top 10 (out of 26) features used by the RF model to distinguish true variants from PTA artifacts. POSITION indicates the base up- (+) or downstream (−) relative to the mutation. (D) Confusion matrix visualizing performance metrics of the RF model in classifying out-of-bag variants. TP, true positive; FN, false negative; FP, false positive; TN, true negative; TPR, true positive rate (sensitivity); TNR, true negative rate (specificity); FDR, false discovery rate; FOR, false omission rate; ACCU, accuracy. (E) The 96-trinucleotide mutational spectra of the base substitutions that were used as PTA artifact or true-positive input for training the RF model (left) and the profiles of the base substitutions that were classified as true or false by the model during cross-validation (right). (F) Heatmap showing the cosine similarities between the base substitutions used in the training set and the base substitutions classified during cross-validation and the previously defined mutational signature of PTA artifacts. (G) Spectra of indels detected in bulk WGS data of AML blasts (top) or before (center) and after (bottom) PTATO filtering of PTA-based WGS data of an HSPC of the same individual. (H) Numbers and types of indels detected before (top) and after (bottom) PTATO filtering in samples analyzed by bulk WGS or PTA-based WGS (highlighted by blue shading). MH, microhomology; ins, insertion; del, deletion.

The artifacts generated by PTA have been shown to follow a specific, non-random 96-trinucleotide mutational profile in WGS data.^17^^,^^18^ We hypothesized that we could use a machine learning approach to distinguish PTA artifacts from true-positive single base substitutions based on multiple genomic features (Figure 1A). For this, we trained a random forest (RF) model, which we previously showed to be highly effective in attributing individual mutations to a specific mutational process.^20^ To generate a confident set of true-positive somatic single base substitutions for training of the classifier, we sequenced 11 samples of three patients with acute myeloid leukemia (AML) and a clonal lymphoblastoid cell line (AHH-1) using regular bulk WGS as well as single-cell WGS after PTA (Figure 1B and Tables S1 and S2). Somatic base substitutions that were shared between the bulk and single-cell sequenced samples were used as high-confidence true variants for training. We combined two approaches to generate a confident set of PTA artifacts for training. First, we PTA amplified and sequenced the genomes of three single umbilical cord blood-derived HSPCs. Most of the unique somatic variants in these cells will be PTA artifacts, because HSPCs at birth only harbor 20–50 somatic mutations.^21^^,^^22^^,^^23^ Second, we selected artifacts from the sequenced AML and cell line PTA samples by implementing and applying a linked read analysis. In this analysis, artifacts are detected because they are not correctly phased with neighboring sequencing reads containing germline variants.^19^ The linked read analysis detects a small subset of artifacts with high specificity, but low sensitivity, as only a minority of variants (10%–27%) can be linked to an informative germline variant.^19^ We varied the ratio between true and false positives in the training set to determine how different ratios affect performance and found that balancing the true and false positives 1:1 yielded the best training results (Figure S1A). In total, 756 PTA artifacts and 756 true-positive single base substitutions were used to train the RF model (Figure 1B).

To train the RF model, we used a variety of 26 different genomic features, such as the level of allelic imbalance of the region the variant is located in, the mutation type, the 10-base pair (bp) sequence context around the variant, the distance to the nearest gene, and replication timing (Figures 1C and S1B). The allelic imbalance is the most important variable in the model (Figures 1C and S1B). This variable is an estimation how well the variant allele frequency (VAF) of a variant matches the modeled pattern of phased VAFs of surrounding germline variants.^21^ Other important features for classifying PTA artifacts are the DNA replication timing of the locus the variant is in, whether the variant is in a repeat region, and the distance of the variant to the nearest gene (Figures 1C and S1B). These features are likely important, because the distribution of true somatic mutations is known to be biased across the genome, such as depleted in early replicating regions and gene bodies.^22^ In contrast, as PTA occurs on naked DNA, PTA artifacts are more randomly distributed over these features in the genome.

The RF model calculates a probability score that a candidate variant is a PTA artifact. As the PTA efficiency and the ratios between true and false positives can vary between samples, a sample-specific cutoff needs to be set above which variants are classified as artifacts. To set an optimal cutoff for each sample, we applied two complementary methods (Figures 1A and S1C–S1G). First, PTATO uses the implemented linked read analysis to classify the small subset of somatic variants that can be linked to informative germline variants as true or false positive. Next, it takes the PTA probability scores for all the variants classified by the linked read analysis and calculates precision-recall curves to determine the optimal cutoff to discriminate these two groups (Figures S1E and S1F). Although this method works well to determine an optimal PTA probability cutoff for most samples, we noted that for some samples, accurate precision-recall curves could not be generated because these samples have too few informative true variants (Figures S1E and S1F). Therefore, we included a second, independent method to determine the PTA probability cutoff by making use of 96-trinucleotide mutational spectra. In this method, a range of increasing potential cutoffs are taken. For each of these potential cutoffs, the mutational spectra are calculated for the variants with PTA probability scores below the specific cutoff (Figure S1G). The mutational spectra at low cutoffs will contain mostly true variants, whereas the mutational spectra at high cutoffs will contain both true variants and artifacts. Hierarchical clustering is used to determine at which cutoff the mutational spectra of the variants passing the filters start to diverge (due to inclusion of artifacts with a different mutational spectrum) from the spectra of the true variants with low PTA probability scores (Figure S1G).

The RF model was predicted to distinguish artifacts from true-positive base substitutions in the out-of-bag sets with an accuracy of 74% (precision = 0.73 and sensitivity = 0.74, Figure 1D) and an area under the curve for precision-recall rates of 0.79 (Figure S1H). Importantly, the 96-trinucleotide mutational spectra of the base substitutions predicted to be false or true variants by PTATO were similar to the profiles of the input PTA artifacts (cosine similarity is 0.99) or true-positive variants (cosine similarity is 0.97), respectively (Figures 1E and 1F).

Compared to the base substitution artifacts, the indel artifacts caused by PTA follow an even more specific pattern, which is mainly characterized by C or T insertions at long homopolymers (repeats of the same nucleotide) (Figures 1G and 1H).^18^ We found that exclusively filtering indel artifacts that are recurrently called in multiple unrelated individuals and filtering insertions at long (5-bp+) homopolymers was even more effective than training an RF model for indel filtering. We created an indel exclusion list containing 5,179,372 indels, which were detected in at least two individuals, across 139 PTA WGS samples of 22 individuals (Figures S2A and S2B). Filtering candidate variants using this list removed most indel artifacts in the samples that were used for training the RF model (Figure S2C), leading to indel burdens and patterns that were comparable (cosine similarity = 0.88) between those found in bulk and PTA-based WGS data (Figures 1G, 1H, and S2D). In contrast to SCAN2, which builds a new indel filter list for every analysis if there are sufficient samples,^18^ PTATO’s approach of using a predefined indel filter list is also applicable to small sets of samples and makes indel filtering more comparable between different analyses. Thus, these initial validations demonstrate that PTATO can accurately discriminate true- and false-positive base substitutions as well as indels using machine learning classification and filtering based on recurrence, respectively.

### Validation of the random forest model

We performed several experiments to test the performance of PTATO on samples that were not used in the training set. First, to assess how well PTATO performs on samples containing different ratios of true- and false-positive base substitutions, we *in silico* mixed different numbers of true base substitutions with a fixed set of PTA artifacts. For this, we collected true somatic base substitutions that were detected in both PTA and bulk WGS samples of two additional AML patients whose samples were not included in the training. Additionally, we obtained PTA artifacts using WGS of an additional PTA-amplified umbilical cord blood sample. This *in silico* analysis showed that the performance of PTATO improves with increasing numbers of true variants, especially if there are more than 200 true base substitutions in a sample (Figures S3A and S3B). Subsequently, to estimate how well PTATO can distinguish true mutations of different mutational backgrounds from PTA artifacts, we *in silico* mutated the trinucleotide sequence context of true-positive base substitutions (while keeping the other features the same) to match the 96-trinucleotide spectra of 54 different mutational signatures. This *in silico* mutagenesis experiment revealed that PTATO can accurately detect mutations of the most commonly occurring mutational signatures (e.g., SBS1, SBS5, and SBS18), but also that accuracy is lower for some less prevalent signatures that are very similar to the PTA artifact signature (e.g., SBS30) (Figure S3C).

**Figure 2:**
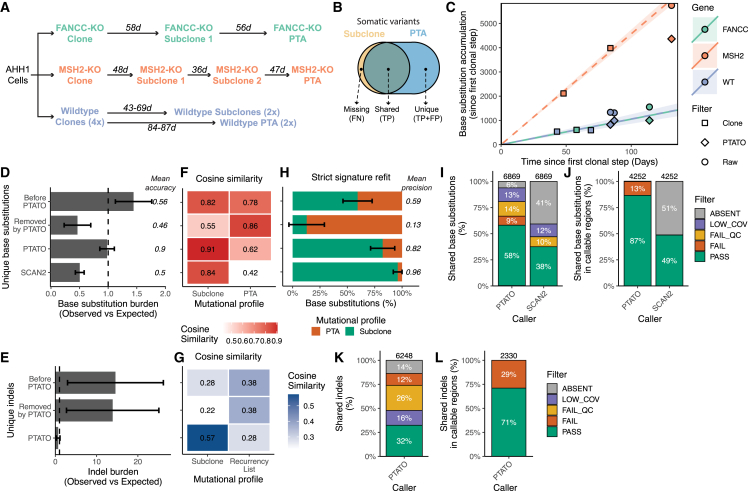
Filtering by PTATO enables accurate analyses of somatic mutation patterns and burdens (A) Schematic overview of the clonal steps performed for the three types of clonal cell lines generated in this study. Numbers indicate the days (d) in culture between the single-cell sorts, which are used to calculate mutation rates for each cell line. (B) Venn diagram indicating which variants were used as false negatives (FN), true positives (TP), and false positives (FP). (C) Accumulation of base substitutions per sample since the first clonal step. The circles and diamonds indicate the number of base substitutions detected in the PTA samples before and after PTATO filtering, respectively. (D) Observed versus expected number of base substitutions in the PTA samples before PTATO filtering, removed by PTATO, after filtering by PTATO and after filtering by SCAN2. Data are represented as the mean (± SEM) in the four PTA samples. (E) Observed versus expected (OE) number of indels in the PTA samples before or after filtering by PTATO and after filtering by SCAN2. Data are represented as the mean (± SEM) in the four PTA samples. Accuracy is determined as the mean absolute difference between the OE values and an OE value of 1. (F) Heatmap showing the mean cosine similarities between the 96-trinucleotide profiles of the unique base substitutions before PTATO filtering, removed by PTATO, after PTATO filtering, or after SCAN2 calling and the profiles of the subclones analyzed by bulk WGS or the previously defined universal PTA artifact signature.^18^. (G) Heatmap showing the mean cosine similarities between the profiles of the unique indels before PTATO filtering, removed by PTATO, or after PTATO filtering and the indel profiles of the subclones analyzed by bulk WGS or the list of recurrent indels used for filtering. (H) Mean contributions (± SEM) of the universal PTA artifact signature and the mutational signatures of the subclones to the mutational profiles in the four PTA samples before PTATO filtering, removed by PTATO, after filtering by PTATO, or after filtering by SCAN2. Precision is determined as the mean contribution of the mutational signatures of the subclones to the mutational profiles of the PTA samples. (I) Fractions of shared base substitutions present in the subclones that are also detected (PASS) in the PTA samples originating from these subclones by PTATO or SCAN2 (SCAN2 could not be used to study indels in these samples). (J) Fractions of base substitutions after excluding the variants (in both the PTATO and SCAN2 call sets) with low coverage (LOW_COV), low genotype quality (LOW_QC), or undetected variants (ABSENT) as determined by PTATO. Few shared variants are (mis)classified as artifact (FAIL) in the PTA samples. (K) Fractions of shared indels present in the subclones that are also detected (PASS) in the PTA samples originating from these subclones by PTATO or SCAN2 (SCAN2 could not be used to study indels in these samples). (L) Fractions of indels after excluding the variants with low coverage (LOW_COV), low genotype quality (LOW_QC), or undetected variants (ABSENT) as determined by PTATO. Some indels are (mis)classified as artifact (FAIL) in the PTA samples (because they are present in the exclusion list or are insertions in long homopolymers).

Secondly, we inactivated the *FANCC* and *MSH2* genes in the human AHH-1 lymphoblastoid cell line using CRISPR-Cas9 gene editing (Figure S4). Inactivation of these genes and their associated DNA repair pathways has been shown to induce various specific base substitution and indel signatures,^23^^,^^24^^,^^25^ enabling us to test the performance of PTATO on a variety of mutational outcomes. We performed several sequential *in vitro* single-cell clonal expansion steps (Figures 2A and 2B), followed by bulk WGS of the expanded (sub)clones, to calculate the mutation rates in these cell lines. Bulk WGS of the subclones showed that the wild-type, *FANCC*^−/−^, and *MSH2*^*−*^/^−^ AHH1 clones acquire respectively 10.6, 10.5, and 52.6 base substitutions and 1.02, 1.12, and 91.1 indels per day in culture on average (Figures S5A and S6A). Subsequently, after further *in vitro* expansion of the subclones (Figure 2A), we sorted single cells of each subclone and performed WGS after PTA. The standard GATK-based somatic variant calling pipeline (STAR Methods) without PTATO filtering detected a 1.37- to 1.86-fold higher base substitution rate (Figures 2C, 2D, and S5A) and a 12- to 29-fold higher indel rate (Figures 2E and S6A–S6C) in the PTA-amplified wild-type and *FANCC*^−/−^ samples compared to the subclones analyzed by bulk WGS. PTATO removed most excess mutations, and the calculated mutation burdens after filtering by PTATO and normalization for the fraction of the genome that was callable (STAR Methods) matched the expected mutation burden (based on extrapolation of the mutation rates determined by bulk WGS of the subclones) with a mean accuracy of 89.5% (Figures 2C–2E, S5A, S5B, and S6A–S6C). In comparison, SCAN2^18^ reported a mutation burden that was on average 50.4% lower than the expected burden (Figure 2D). Filtering by PTATO also improved the similarity between the mutational profiles of the PTA-amplified samples and the profiles of the corresponding bulk WGS-analyzed subclones (Figures 2F, 2G, S5C–S5G, S6D, and S6E). The exact number of PTA artifacts in these PTA samples is not known. Therefore, to estimate the number of PTA artifacts before and after PTATO filtering, we performed a bootstrapped mutational signature refit against the mutational profiles of the PTA artifacts and the subclones sequenced with regular WGS. This analysis showed that PTATO improved the precision of base substitution filtering over standard GATK-based somatic variant filtering from 59% to 82%, which is only modestly lower (14.6%) than the 96% precision that SCAN2 showed for these samples (Figure 2H). As shown for the *MSH2*^−/−^ cell sequenced after PTA, PTATO can also accurately remove PTA artifacts from samples with low amplification quality (Figure S6F), although the sensitivity to detect true variants is reduced due to uneven coverage and loss of heterozygosity over the genome (Figures S5 and S6).

The somatic variants detected in the (sub)clones should also be present in the corresponding PTA-amplified samples derived from those (sub)clones and thereby should form a reliable set of true-positive variants. Between 45% and 69% of the base substitutions (Figure 2I) and 31%–56% of the indels (Figure 2K) that were detected in the (sub)clones were also reported in the PTA-amplified cells after PTATO filtering. The clonal variants absent in the PTA-amplified cells were mainly missed due to low coverage and allelic dropout (Figures 2I and 2K), predominately indicating a limitation of the PTA reaction instead of incorrect filtering by PTATO. Importantly, only 10%–16% of the base substitutions and 29% of the indels found in both the (sub)clones and the PTA-amplified cells were classified as a PTA artifact by PTATO, showing that PTATO has a mean sensitivity of 86.8% in discriminating detectable true single base substitutions from artifacts in callable loci (Figures 2J and 2L). In contrast, SCAN2 reported on average only 48.8% of these base substitutions shared between these PTA-amplified cells and bulk WGS-analyzed (sub)clones in the callable fractions of the genomes (∼78% less than PTATO, Figure 2J). This finding is in line with the ∼46% sensitivity reported for this tool.^18^ Indels could not be assessed by SCAN2 for these samples, because that required more PTA samples in a single analysis to build a cross-sample filter list. This finding underscores the practicality of PTATO’s use of a predefined indel exclusion list instead of creating a novel filter list for each separate analysis.

Thirdly, we further validated the performance of PTATO by applying it to a previously published PTA-based WGS dataset of human umbilical cord blood cells that were treated with a vehicle (VHC) control or with different dosages of the mutagens D-mannitol (MAN) or *N*-ethyl-*N*-nitrosourea (ENU)^17^ (Figure S7). We performed strict mutational signature refitting to the universal PTA artifact signature^18^ and the SBS1, SBS5, and ENU-associated^26^ signatures to estimate respectively the number of false- and true-positive base substitutions before and after filtering. This analysis showed that filtering by PTATO removed most variants associated with the mutational signature of PTA artifacts with a mean estimated precision of 92% while keeping most single base substitutions associated with signature SBS5 and/or the ENU-associated signature^26^ (Figures S7B–S7E). In the samples treated with a high dose of ENU resulting in a high mutation burden, PTATO detected SBS5- and ENU-associated mutations with an estimated sensitivity of 89% (compared to 60% for SCAN2) (Figures S7D and S7E). The estimated sensitivity to detect true mutations dropped in the VHC-treated control sample with low mutation burden to 37% (compared to 4% for SCAN2) (Figures S7D and S7E). In total, SCAN2 detected 35% less SBS5- and ENU signature-related base substitutions (Figure S7D). Additionally, the 96-trinucleotide profiles detected by SCAN2 in the VHC samples matched the universal PTA artifact signature with high cosine similarity (0.89 compared to 0.6 for PTATO), suggesting it mostly detected artifacts in these samples (Figures S7C).

Finally, to test how the RF model of PTATO performs on non-hematological samples, we isolated five single cells from a clonal intestinal organoid culture and performed PTA, WGS, and PTATO analysis on these cells (Figure S8). Refitting the 96-trinucleotide spectra against the universal PTA artifact signature^18^ and a previously described signature of somatic base substitutions accumulating in intestinal organoids *in vitro* (Figure S8C)^6^ showed that PTATO can also adequately remove PTA artifacts from single-cell PTA data of intestinal organoids (Figure S8D).

These validations show that PTATO can effectively filter single base substitutions and indel artifacts from PTA-based WGS data from different sources, enabling accurate analyses of somatic mutational burdens, patterns, and signatures in single cells.

### Unaltered patterns of indels in most HSPCs of patients with FA

To study the consequences of inactivation of the FA DNA repair pathway in human HSPCs *in vivo*, we aimed to analyze the genomes of HSPCs of multiple individuals with FA. However, although we flow sorted at least 200 single HSPCs of each of six patients for *in vitro* clonal expansion, only for two patients a limited number of clones (one and eight, respectively) expanded to a size large enough for bulk WGS, underlining the need for direct single-cell WGS. Therefore, we used PTA followed by PTATO analysis to study the genomes of single HSPCs derived from bone marrow aspirates of five different individuals with FA (Table 1). In addition, we analyzed the genomes of bulk AML blasts and three PTA-amplified (pre-)leukemic stem cells from a patient with FA (IBFM35) who developed AML after a failed hematopoietic stem cell transplantation.

**Table 1:** FA patient characteristics at moment of bone marrow puncture

Individual	Age (years)	Affected Fanconi anemia gene	Fanconi anemia driver mutations	HSC clones	Bone marrow cellularity	Hematological status	Cytogenetic aberrations
PMCFANC01^a^	7.9–8.4	*FANCC*	c.67delG; c.67delG	1	moderate/low	normal/mild cytopenia	none
PMCFANC02	15.9	*FANCD1/BRCA2*	c.5213_5216delCTTA; c.9302T>G	8	moderate	normal	none
PMCFANC03	15	*FANCA*	c.1361_1370delCCTCCTTTGG; c.1361_1370delCCTCCTTTGG	0	low	mild cytopenia	none
PMCFANC06	17	*FANCA*	c.67delG; c.67delG	0	moderate	normal	none
PMCFANC08	10.3	*FANCA*	c.2151+1dup; c.2121delC	0	moderate	mild cytopenia	none
IBFM35	14.8	*FANCA*	c.3639delT; c.3639delT	0	N/D	AML	N/A

a Bone marrow aspirates from PMCFANC01 were collected at two different time points. HSC, hematopoietic stem cell.

**Figure 3:**
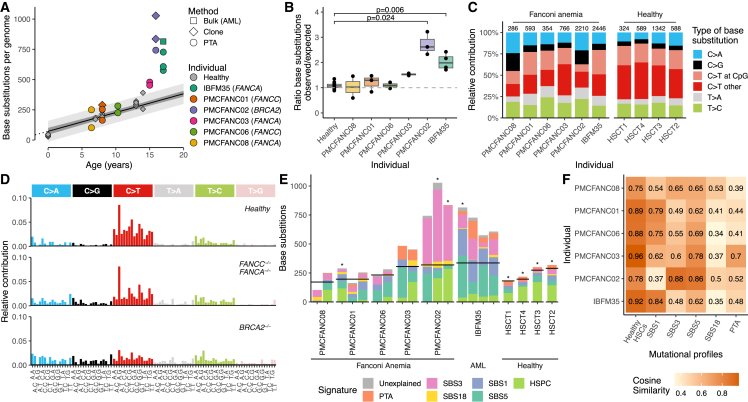
PTATO detects normal single base substitution burdens in most human FA HSPCs (A) Correlation of the number of somatic single base substitutions per HSPC genome of healthy donors (gray points) and patients with FA. Linear mixed modeling showed that healthy HSPCs accumulate base substitutions in a linear fashion with age.^27^^,^^28^ The 95% confidence interval and the prediction interval of the model are indicated by the dark gray and light gray shading, respectively. (B) Ratios between the observed and expected number of base substitutions per genome (sorted on age) based on extrapolation of the age linear mixed model. To match the ages of the patients with FA, only 12 HSPCs of four healthy donors (HSCT1–4, ages 7 to 14) are included in this and following panels. Adjusted p values indicate multiple testing corrected significant differences (p_adj_ < 0.05) between three FA patients and the age-matched healthy donors (Bonferroni-corrected Wilcoxon Mann-Whitney test). (C) Mutation spectra showing the relative contribution of each base substitution type in the genomes of the donors. Numbers above the bar indicate the total number of base substitutions found in the samples from each individual. (D) The averaged 96-trinucleotide mutational profiles of the HSPCs of the four healthy individuals (HSCT1–4), the patients with mutations in *FANCA* or *FANCC* (PMCFANC01, PMCFANC03, PMCFANC06, PMCFANC08), and the patient with mutations in *BRCA2* (PMCFANC02). (E) Contribution of base substitution mutational signatures commonly found in blood cells^27^^,^^28^ to each FA sample or healthy individual (averaged). Horizontal black lines indicate the expected number of base substitutions based on age. Non-PTA samples sequenced with bulk WGS are indicated by an asterisk. For donors HSCT1 to HSCT4, the mean contributions over all samples per donor is shown. (F) Cosine similarities between the mean 96-trinucleotide mutational profiles of the HSPCs of FA patients with the profiles of the healthy HSPCs from the four age-matched donors and the mutational signatures.

First, we compared the PTATO-filtered base substitutions detected in the HSPCs of individuals with FA with previously generated WGS data of 34 clonally expanded HSPCs of 11 healthy donors.^27^^,^^28^ This comparison showed that most of the FA HSPCs had similar somatic single base substitution burdens (Figures 3A, 3B, S9A, and S9B), patterns (Figures 3C and 3D), and signatures (Figures 3E and 3F) as HSPCs of healthy individuals. Patient PMCFANC02, whose FA was caused by biallelic germline variants in the *FANCD1*/*BRCA2* gene, and AML patient IBFM35 formed exceptions with respectively 3-fold and 2-fold higher somatic base substitution burden than expected for their age (Figures 3A and 3B). The elevated mutation burden in PMCFANC02 is mostly caused by base substitutions characterized by mutational signature SBS3, which is associated with homologous recombination deficiency^29^^,^^30^ and which is barely detected in the other FA patients (Figures 3E and 3F).

**Figure 4:**
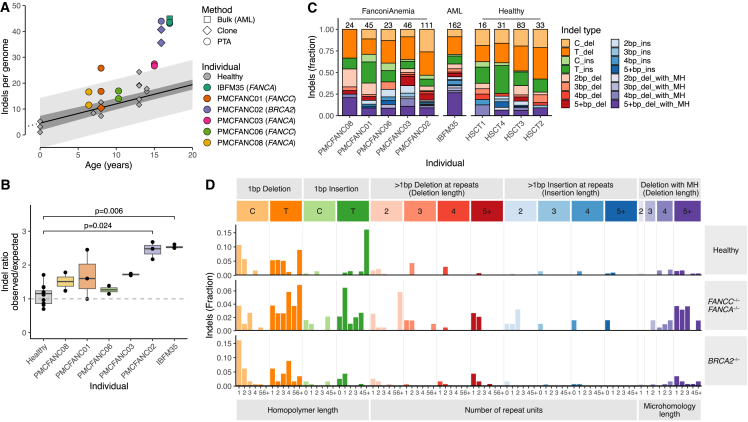
Small insertions and deletions in HSPCs of patients with FA (A) Correlation of the number of somatic indels per HSPC genome of healthy donors (gray points) and patients with FA. Linear mixed modeling showed that healthy HSPCs accumulate indels in a linear fashion with age.^27^^,^^28^ The 95% confidence interval and the prediction interval of the model are indicated by the dark gray and light gray shading, respectively. (B) Ratios between the observed and expected number of indels per genome (sorted on age) based on extrapolation of the age linear mixed model. To match the ages of the patients with FA, only 12 HSPCs of four healthy donors (HSCT1–4, ages 7 to 14) are included in this and following panels. p values indicate multiple testing corrected significant differences (p_adj_ < 0.05) between two of the FA patients and the age-matched healthy donors (Bonferroni-corrected Wilcoxon Mann-Whitney test). (C) Indel spectra showing the relative contribution of the main indel types in the genomes of the donors. Numbers above the bar indicate the total number of indels found in the samples from each individual (without extrapolation for callable loci). (D) Total averaged indel profiles of the HSPCs of the four healthy individuals (HSCT1–4), the patients with mutations in *FANCA* or *FANCC* (PMCFANC01, PMCFANC03, PMCFANC06, PMCFANC08), and the patient with mutations in *BRCA2* (PMCFANC02).

Subsequently, we compared the somatic indel accumulation between HSPCs of patients with FA and healthy bone marrow donors. Only patients PMCFANC02 (*FANCD1*/*BRCA2*) and IBFM35 (*FANCA* and AML) had a significantly increased indel burden compared to healthy HSPCs (also in their bulk-sequenced clones and leukemic blasts) (Figures 4A, 4B, and S9C). The relatively high indel burdens in the HSPCs of these two patients did not seem to be caused by a specific type of indel (Figures 4C and 4D). These findings, which are in line with observations in FA mouse models^12^ and FA cell lines,^23^ confirm that PTATO-based filtering of PTA-based WGS data can be used to accurately study somatic mutations in single cells that cannot be clonally expanded *in vitro*.

### Accurate detection of structural variants in PTA-based sequencing data

**Figure 5:**
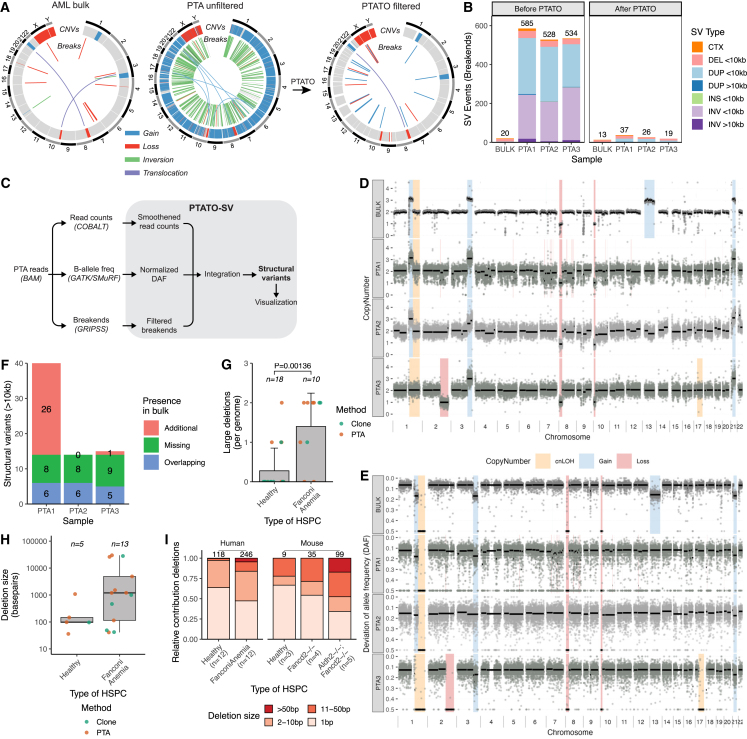
SV filtering by PTATO reveals an increased deletion burden in HSPCs of patients with FA (A) Circos plots showing copy number variants (CNVs) and balanced SVs in a PTA (left/center) and bulk WGS sample (right) of patient IBFM35. The standard SV calling pipeline for bulk WGS generates hundreds of false-positive calls in PTA samples (left), most of which are removed by PTATO filtering (center), leading to similar SV profiles as a sample sequenced by bulk WGS (right panel). (B) Number of SV events detected by GRIDSS without filtering by PTATO (left) and the number of SVs remaining after filtering by PTATO (right) in bulk and PTA-based WGS samples of IBFM35. (C) Schematic overview of the SV calling and filtering strategy tailored for PTA-based WGS data implemented in the PTATO pipeline. (D) Copy number profiles (100-kb windows) of the AML bulk sample analyzed by the bulk WGS SV calling pipeline and three PTA samples analyzed by PTATO. Background shadings indicate the final copy number call made by PTATO (for PTA samples) or PURPLE (for the bulk WGS sample). (E) Deviation of allele frequency (DAF) plots (100-kb windows) of the AML bulk sample and three PTA samples. The DAF depicts the absolute difference between 0.5 (perfect heterozygosity) and the actual allele frequency of a germline variant. (F) Number of SVs (>10 kb in size) that are present in the HSPCs and present (“Overlapping”) or absent (“Additional”) in the AML bulk or present in the bulk but absent in the HSPCs (“Missing”). (G) Number of deletions (>25 bp) detected by GRIDSS and PTATO in genomes of HSPCs of FA patients or healthy donors (including five cord blood samples sequenced after PTA). Numbers shown above the bars indicate the number of individuals per group. The p value was calculated by Wilcoxon Mann-Whitney test. (H) Size (in bp) of each detected deletion in HSPCs of healthy donors and patients with FA (no significant difference Wilcoxon Mann-Whitney test). Numbers above the boxes indicate the total number of deletions per group. (I) Distribution of the sizes of small (detected by GATK for the human samples) and large (detected by GRIDSS for the human samples) deletions in human and mice^12^ HSPCs with different genetic backgrounds. The numbers above the bars indicate the total number of deletions analyzed per group.

It has been shown that HSPCs of FA mouse models^12^ and leukemias^31^ and squamous cell carcinomas^32^ of human patients with FA have high burdens of somatic SVs. Existing bioinformatic tools for single-cell WGS are usually limited to the detection of copy number changes based on read depth,^33^ and we found that more comprehensive SV calling pipelines for bulk WGS data detect many false-positive variants in PTA-based data (Figures 5A and 5B). To study somatic SVs in the HSPCs of the patients with FA, we needed to optimize an SV calling and filtering approach specifically designed for PTA-based WGS data. PTATO integrates calling of SVs by GRIDSS^34^ and COBALT^35^ based on read depth, B-allele frequencies, split reads, and discordant read pairs followed by various normalization and filtering steps tailored for PTA-based WGS data (Figures 5C, S10 and S11).

First, copy number variant (CNV) calling by PTATO started with calculating and segmenting the read coverage over the genome (Figures S10C and S10D). We noted that the local fluctuations in coverage profiles are recurrent between PTA samples (Figures S10A and S10B). Therefore, we collected copy number profiles of 12 copy number neutral PTA samples and created a panel of normals (PON) to smoothen the coverage in test samples (Figures S10E–S10J). To determine which genomic segments are potential copy number gains or losses, PTATO determined if the smoothened read coverage within a segment is significantly divergent from (1) the coverage of other segments within the same sample and (2) the mean coverage of the same genomic segment in the PON samples (Figure S10H).

Second, the ability to accurately detect germline base substitutions in PTA-based WGS data enabled PTATO to use the B-allele frequencies of germline variants to complement CNV calling (Figures S11A and S11B). PTATO minimized the noise in allele frequencies of germline variants by binning multiple germline variants (Figures S11A and S11B). This B-allele frequency information is integrated with the coverage profiles to determine which genomic segments are copy number losses, gains, or copy number neutral loss-of-heterozygosity (cnLOH) regions (Figures 5C, 5D, S11A, and S11B).

Finally, the relatively even coverage over the genome in PTA data enabled the detection of split reads and discordant read pairs (break-ends). Hundreds to thousands of artificial SVs, mainly small events that were called as inversions or duplications, were detected in PTA samples by the standard SV calling pipeline (Figures 5A and 5B). PTATO filtered these raw calls using a recurrence list, by excluding SV calls with only one breakpoint junction and by excluding inversion calls that are less than 1 kb in size.

We applied the SV filtering to PTA-based WGS data of three HPSCs of a patient with AML (IBFM35) to compare the SV calls in these cells with the SVs detected in the bulk AML sample of this patient. PTATO removed most excess SV calls (Figures 5A and 5B) and determined accurate copy number profiles for these samples (Figures 5D and 5E). Not all SVs present in the AML bulk sample were detected in the PTA samples (Figure 5H). Some SVs (such as the t(3; 10) translocation) were missing in the PTA samples due to low coverage around the breakpoints or due to imbalanced amplification (Figure 5A). However, several SVs (such as the gain of chromosome 13) were not detected in any of the single HSPCs despite proper amplification and coverage of these regions, suggesting that these HSPCs are non- or pre-leukemic cells (Figures 5D–5F). To further test PTATO’s SV pipeline, we applied it to PTA-based WGS data of an HSPC of AML patient IBFM26 and two single AHH-1 cells. Also in these cells, PTATO generated copy number profiles that were similar to those obtained after bulk WGS and PTATO accurately detected the known copy number gains and loss (Figures S11C and S11D).

After optimization of SV detection in PTA-based WGS data, we looked for the presence of somatic SVs in the HSPCs of the other patients with FA. We did not observe any large chromosomal abnormalities or translocations (Figure S12). However, we observed 13 deletions with read depth, B-allele frequency (if overlapping germline variants), and split read/discordant read pair support in the 10 cells with sufficient quality (two cells had insufficient quality for accurate CNV detection, Figure S12) ranging from 41 to 29,850 bp (Figures 5G–5I and Table S3). The deletions were detected in both the PTA-amplified HSPCs as well as the clonally expanded HSPCs, indicating that the detected deletions are probably not artifacts. Additionally, we rarely observed deletions larger than 100 bp in the healthy HSPCs sequenced after clonal expansion or PTA, further supporting that there is an increased burden of deletions in HSPCs of FA patients (Figures 5G–5I).

## Discussion

The introduction of PTA greatly improved the accuracy of single-cell WGA, leading to rapid adoption in the field.^17^^,^^18^^,^^36^^,^^37^^,^^38^ However, bioinformatic tools making optimal use of the potential of PTA have been lacking. To address this, we developed the PTATO pipeline that can accurately distinguish true-positive single base substitutions, indels, and SVs from false-positive artifacts in PTA-based WGS data. The main benefit of PTATO over other tools, in addition to SV filtering, is the relatively high sensitivity between 70% and 89% (compared to ∼46% reported by SCAN2) to distinguish true base substitutions from artifacts in the callable genome. This means that less extrapolation is required to estimate the true somatic mutation burden in cells, which may be especially important for driver mutation detection and retrospective lineage tracing experiments. The RF model used here was trained and tested mainly on hematological samples, but we showed that it can also effectively remove PTA artifacts from other cell types such as intestinal organoid samples. Nevertheless, if necessary, the RF model included in PTATO can be easily retrained (e.g., by altering the sequence contexts of the true-positive variants in the training set as in Figure S3C), making it a flexible tool.

We demonstrated the performance of PTATO by analyzing the genomes of single HSPCs of patients with FA, which could not be clonally expanded *in vitro* for bulk WGS. This analysis showed that most HSPCs of patients with FA have similar somatic mutations burdens as HSPCs of healthy donors but with an increased number of deletions. These results are in line with findings in mouse models^12^ and cell lines^23^ of FA. Furthermore, the patterns of SVs detected in the HSPCs of FA patients (mostly deletions <100 kb) are similar to the SV patterns found in leukemias^31^ and head and neck cancers^32^ of patients with FA. The increased deletion burden suggests an increased occurrence of double-stranded breaks and/or incorrect repair of these breaks in FA HSPCs, which fits with the molecular functions of the FA DNA repair pathway.^8^ It is likely that there is selection against HSCs with more genomic rearrangements without the necessary driver mutations to survive, leading to a gradual depletion of such HSCs in FA patients. The analyzed HSPCs of one FA patient with germline *FANCD2*/*BRCA2* mutations showed strongly elevated somatic mutation rates, which is consistent with the broader role of BRCA2 independent of the FA DNA repair pathway.^39^ This also highlights that the phenotypic heterogeneity between FA patients may be accompanied by genomic heterogeneity in HSPCs between patients.^40^ Further studies including larger patient cohorts are required to characterize this genomic heterogeneity, which is likely dependent on the causative germline mutations and disease progression stage.

We showed that our PTATO filtering approach improves the usability of PTA, further narrowing the gap in data quality between single-cell WGS and regular bulk WGS. This will be especially important for the genomic analyses of cells that cannot be clonally expanded for regular WGS, such as diseased or differentiated cells. The accurate characterization of single-cell whole genomes by PTA followed by PTATO analysis enables the study of ongoing mutational processes in tissues and cancers, because this combined approach is not limited to analysis of relatively early, clonal mutations like regular bulk WGS.^41^ We foresee that such single-cell genome analyses made possible by PTATO will yield an unprecedented view of tumor heterogeneity and cancer evolution.

### Limitations of the study

PTATO can detect base substitutions and indels with higher sensitivity (70%–89% for callable genomic loci) than other tools like SCAN2 with similar precision (70%–92%). The accuracy of somatic variant filtering is generally lower in samples with relatively low mutation burdens (<200 somatic base substitutions) compared to samples with higher burdens, but also for such samples, PTATO is more effective in removing PTA artifacts than SCAN2. This illustrates the general challenge to filter mutations in single cells with low mutation burdens such as umbilical cord blood samples, but most cells have more than 200 somatic variants. We note that in some of the analyses performed to determine the performance of PTATO, the exact number of PTA artifacts was unknown. In some of these experiments, we therefore relied on a mutational signature refit to estimate the number of PTA artifacts in a sample, which is less accurate than using a golden truth set of PTA artifacts. Our strategy to remove indel artifacts based on recurrence and presence in long homopolymers is highly effective in removing PTA indel artifacts, but it also excludes some true indels (including some potential disease-causing indels) that are present in bulk WGS samples (Figure 1H). Finally, PTATO enables SV filtering of PTA-based WGS data. Most SV artifacts are removed by PTATO, but the accuracy of SV detection is dependent on the quality of the PTA reaction. Samples with a relatively low DNA output after PTA may show noisy copy number profiles and large regions of loss of heterozygosity due to uneven amplification of the alleles. PTATO calculates quality control metrics to identify such samples with low amplification quality. Precise calculation of performance metrics (e.g., sensitivity and precision) of SV detection by PTATO will require more WGS data of PTA and bulk samples containing the same SVs.

## STAR★Methods

### Key resources table

**Table:** 

REAGENT or RESOURCE	SOURCE	IDENTIFIER
**Antibodies**		

Rabbit monoclonal anti-MSH2 (D24B5)	Cell Signaling Technology	Cat.#2017S; RRID:AB_2235387
Mouse monoclonal anti-α-Tubulin (B-5-1-2)	Sigma-Aldrich	SKU T5168-100UL; RRID:AB_477579
Goat polyclonal anti-rabbit IgG IRDye 800CW	Li-Cor	P/N 926–32211; RRID:AB_621843
Goat polyclonal anti-mouse IgG IRDye 680RD	Li-Cor	P/N 926–68070; RRID:AB_10956588
Anti-human CD34-BV421 (clone 561)	BioLegend	Cat.#343610; RRID:AB_2561358
Anti-human Lineage Cocktail (CD3/CD14/CD19/CD20/CD56)-FITC (clones UCHT1, HCD14, HIB19, 2H7, HCD56)	BioLegend	Cat.#348801; RRID:AB_10612570
Anti-human CD38-PE (clone HIT2)	BioLegend	Cat.#303506; RRID:AB_314358
Anti-human CD90-APC (clone 5E10)	BioLegend	Cat.#328114; RRID: AB_893431
Anti-human CD45RA-PerCP/Cy5.5 (clone HI100)	BioLegend	Cat.#304122; RRID: AB_893357

**Chemicals, peptides, and recombinant proteins**		

Mitomycin C from *Streptomyces caespitosus*	Sigma-Aldrich	SKU M4287-2MG

**Critical commercial assays**		

ResolveDNA Whole Genome Amplification Kit	BioSkryb	100545
QIAamp DNA Micro Kit	QIAGEN	Cat.#56304
DNeasy Blood & Tissue Kit	QIAGEN	Cat.#69506

**Deposited data**		

Raw whole genome sequencing data	This paper	EGA: EGAS00001007288
Processed somatic variant data and western blots	This paper	Mendeley Data: https://doi.org/10.17632/c3r9chw9rb.1
Single-cell PTA-based WGS data from cord blood tissue	Gonzalez-Pena et al.^17^	SRA: SRP178894

**Experimental models: Cell lines**		

Human: AHH-1	ATCC	CRL-8146; RRID:CVCL_3640

**Oligonucleotides**		

Guide RNA *FANCC*: 5′-GCAAGAGATGGAGAAGTGTA-3′	This paper	N/A
Guide RNA *MSH2*: 5′-GTGCCTTTCAACAACCGGTTG-3′	This paper	N/A

**Recombinant DNA**		

Plasmid: pSpCas9(BB)-2A-GFP (PX458)	Ran et al.^42^	Addgene: #48138

**Software and algorithms**		

PTATO	This paper	https://github.com/ToolsVanBox/PTATO; https://doi.org/10.5281/zenodo.8098608
SMuRF v3.0.1	This paper	https://github.com/ToolsVanBox/SMuRF
IAP v2.8.0	University Medical Center Utrecht	https://github.com/UMCUGenetics/IAP
GRIDSS-PURPLE-LINX pipeline v1.3.2	Hartwig Medical Foundation	https://github.com/hartwigmedical/gridss-purple-linx
SCAN2	Luquette et al.^18^	https://github.com/parklab/SCAN2
BWA v0.7.17	Li et al.^43^	https://github.com/lh3/bwa
GATK v4.1.3.0	DePristo et al.^44^	https://gatk.broadinstitute.org/hc/en-us
Picard v2.24.1		http://broadinstitute.github.io/picard
Samtools v1.9.4	Danecek et al.^45^	https://www.htslib.org/
Sambamba v0.8.2	Tarasov et al.^46^	https://github.com/biod/sambamba
Bedtools v.2.30.0	Quinlan and Hall^47^	https://bedtools.readthedocs.io/en/latest/
Nextflow v21.10.6.5661	Di Tommaso et al.^48^	https://www.nextflow.io/
GRIDSS2 v2.13.2	Cameron et al.^34^	https://github.com/PapenfussLab/gridss
GRIPSS	Priestley et al.^35^	https://github.com/hartwigmedical/hmftools/tree/master/gripss
COBALT v1.11	Priestley et al.^35^	https://github.com/hartwigmedical/hmftools/tree/master/cobalt
ShapeIt v4.2.2	Delaneau et al.^49^	https://odelaneau.github.io/shapeit4/
Circos v0.69-9	Krzywinski et al.^50^	http://circos.ca/
MongoDB		https://www.mongodb.com/
ggplot2 v3.4.1	Wickham^51^	https://ggplot2.tidyverse.org/
ggpubr v0.6.0		https://CRAN.R-project.org/package=ggpubr
rstatix v0.7.2		https://CRAN.R-project.org/package=rstatix
MutationalPatterns v3.6.0	Manders et al.^52^	https://bioconductor.org/packages/release/bioc/html/MutationalPatterns.html
VariantAnnotation v1.42.1	Obenchain et al.^53^	https://bioconductor.org/packages/release/bioc/html/VariantAnnotation.html
StructuralVariantAnnotation v1.12	Cameron and Dong 2023^34^	https://www.bioconductor.org/packages/release/bioc/html/StructuralVariantAnnotation.html
BSgenome.Hsapiens.UCSC.hg38 v1.4.4	Pagès2023	https://bioconductor.org/packages/release/data/annotation/html/BSgenome.Hsapiens.UCSC.hg38.html
randomForest v4.7–1.1		https://cran.r-project.org/package=randomForest
Copynumber v1.36	Nilsen, Liestoel and Lingjaerde	https://bioconductor.org/packages/release/bioc/html/copynumber.html
LaplacesDemon v16.1.6		https://CRAN.R-project.org/package=LaplacesDemon
Seqkit v2.2.0	Shen et al.^54^	https://bioinf.shenwei.me/seqkit/
ggeffects v1.1.0	Lüdecke^55^	https://strengejacke.github.io/ggeffects/
TIDE	Brinkman et al.^56^	https://tide.nki.nl/
IGV	Robinson et al.^57^	https://software.broadinstitute.org/software/igv/

**Other**		

Custom code to create figures	This paper	https://github.com/ProjectsVanBox/PTATO; https://doi.org/10.5281/zenodo.8186323

### Resource availability

#### Lead contact

Further information and requests for resources and reagents should be directed to and will be fulfilled by the lead contact, Ruben van Boxtel (R.vanBoxtel@prinsesmaximacentrum.nl).

#### Materials availability

This study did not generate new unique reagents.

### Experimental model and study participant details

#### Human subjects

Bone marrow samples were obtained from the biobank of the Princess Máxima Center for Pediatric Oncology with ethical approval under proposal PMCLAB2018-007 and PMCLAB2019-027. Written informed consents from the included individuals were obtained by the Princess Máxima Center. The use of material for this study was approved by the Biobank and Data Access Committee of the Princess Máxima Center. The umbilical cord blood sample of donor CB15 was obtained via the University Medical Center Utrecht (UMCU). The collection of cord blood samples was approved by the Biobank Committee of the UMCU (protocol number 19–737). Informed consent for these samples was obtained by the UMCU. The samples from IBFM26 and IBFM35 were obtained from the German Society of Pediatric Oncology and Hematology (GPOH), who also obtained informed consent from these individuals. Details about the sex and age of the included sample donors can be found in Table S1.

#### Culture of primary human HSPCs

HSPCs sorted for clonal expansion were cultured in HSPC culture medium for 4 to 7 weeks at 37°C in 5% CO_2_ before collection. HSPC culture medium consisted of StemSpan SFEM medium (STEMCELL Technologies) supplemented with SCF (100 ng/mL), FLT3 ligand (100 ng/mL), IL6 (20 ng/mL), IL3 (10 ng/mL), TPO (50 ng/mL), UM729 (500 nmol/L), and Stemregenin (750 nmol/L). Additionally, mesenchymal stromal cells (MSCs) were cultured from a fraction of bone marrow aspirates by plating cells in 12-well culture dishes with DMEM-F12 medium (Thermo Fisher Scientific) supplemented with 10% fetal bovine serum. The medium was refreshed every other day to remove nonadherent cells, and MSCs could be harvested when confluent (after approximately 2–3 weeks).

#### Generation of gene knockouts in AHH-1 cells

Human B-lymphocyte AHH-1 (CRL-8146) cells (male) were purchased from ATCC. Cells were cultured in RPMI 1640 GlutaMAX medium (Thermo Fisher Scientific) supplemented with 1% Penicillin-Streptomycin (Thermo Fisher Scientific) and 10% horse serum (Thermo Fisher Scientific). Guide RNAs (*FANCC*: 5′-GCAAGAGATGGAGAAGTGTA-3′ and *MSH2*: 5′-GTGCCTTTCAACAACCGGTTG-3′) were cloned into pSpCas9(BB)-2A-GFP (PX458) vector (Addgene #48138).^42^ AHH-1 cells were transfected using Lipofectamine 2000 (Thermo Fisher Scientific). One to two days after transfection, GFP-positive transfected cells were single-cell sorted for clonal expansion on a SH800S Cell Sorter (Sony), which was also used for subsequent clonal steps.

MSH2 inactivation was confirmed using Western blot, Sanger sequencing and WGS. The following antibodies were used for western blotting: rabbit anti-MSH2 (D24B5, 1:2000, Cell Signaling Technology) and mouse anti-α-Tubulin (T5168, 1:5000, Sigma-Aldrich). Anti-rabbit IgG IRDye 800CW (1:10000, Li-Cor) and anti-mouse IgG IRDye 680RD (1:10000, Li-Cor) were used as secondary antibodies. Western blots were imaged on an Odyssey DLx imaging system (Li-Cor).

FANCC inactivation was validated by Sanger sequencing, WGS and MMC sensitivity assay. TIDE^56^ analysis of the Sanger sequencing traces was performed to estimate indel frequencies in the *FANCC* alleles in the edited cells. For the MMC assay, 5000 cells were plated per well (96-well plates) containing 100μL medium supplemented with different concentrations (0, 5, 10, 50, 100, 500 and 100 nM) of MMC (Sigma-Aldrich) in triplicate. After 5 days of incubation, cell survival was measured using the CellTiter-Glo Luminescent Cell Viability Assay (Promega) according to the manufacturer’s protocol.

For the *MSH2*^−/−^ clonal line, two additional consecutive clonal steps were performed (after 48 and 36 days in culture, respectively), and single cells were sorted for PTA 47 days after the third clonal step (Figure 2A). For the *FANCC*^−/−^ clonal line, a second clonal step was performed 58 days after the first clonal step, and PTA was performed 56 days after the second clonal step (Figure 2A). Four clonal lines were generated for the wildtype cells (Figure 2A). From these four clones, two underwent an additional clonal step (43 and 69 days after the first clonal step) and two were single cell sorted for PTA (84 and 87 days after the clonal step). Cells were harvested for DNA extraction when (sub-)clonal lines were sufficiently expanded after single cell sorts.

#### Intestinal organoid culture

The clonal wild-type human intestinal organoid line ASC-5a from donor STE0072 (female) was derived in a previous study.^6^ Intestinal organoids were cultured as previously described^58^ in 10 μL domes of Cultrex Pathclear Reduced Growth Factor Basement Membrane Extract (BME) (3533–001, Amsbio) in growth medium consisting of Advanced DMEM/F12 (Gibco), 1× B27, 1× glutamax, 10 mmol/L HEPES, 100 U/ml penicillin-streptomycin (all Thermo Fisher), 1.25 mM N-acetylcysteine, 10 μM nicotinamide, 10 μM p38 inhibitor SB202190 (all Sigma-Aldrich) and the following growth factors: 0.5 nM Wnt surrogate-Fc fusion protein, 2% noggin conditioned medium (both U-Protein Express), 20% Rspo1 conditioned medium (in-house), 50 ng/mL EGF (Peprotech), 0.5 μM A83-01, and 1 μM PGE2 (both Tocris). For the last two passages, organoids were cultured in medium without antibiotics for 4 days. They were exposed to 0.05% (w/v) FastGreen dye (Sigma) apically, and 5 μg/mL of gentamicin (Sigma) for three days. Primocin (1X, InvivoGen) was added for three days prior to passage or single cell isolation. Single cells were isolated for PTA by dissociating organoids with TrypLE express (Gibco) followed by fluorescence-activated cell sorting (FACS) on an SH800S Cell Sorter (Sony).

### Method details

#### Flow cytometry

Lin^−^ CD34^+^ HSPCs were single-cell sorted by fluorescence-activated cell sorting (FACS) on an SH800S Cell Sorter (Sony) for clonal expansion or PTA. The following antibodies were used for staining: CD34-BV421 (clone 561, 1:20), lineage (CD3/CD14/CD19/CD20/CD56)-FITC (clones UCHT1, HCD14, HIB19, 2H7, HCD56, 1:20), CD38-PE (clone HIT2, 1:50), CD90-APC (clone 5E10, 1:200) and CD45RA-PerCP/Cy5.5 (clone HI100, 1:20). AML blasts were selected based on diagnostic immunophenotyping data if available. In most cases, these blasts were CD33, CD38, and/or CD34 positive. All FACS antibodies were obtained from BioLegend.

#### PTA, DNA isolation and WGS

PTA was performed using the ResolveDNA Whole Genome Amplification Kit (BioSkryb Genomics) according to the manufacturer’s protocol. Instead of 10 min cell lysis on ice as indicated in the protocol, lysis was performed by 5 min incubation on ice followed by 5 min incubation at room temperature to maximize DNA denaturation as previously described.^36^ DNA samples from bulk AML and bulk MSCs (for germline control) were isolated using the QIAamp DNA Micro Kit (QIAGEN) or DNeasy Blood & Tissue Kit (QIAGEN) according to the manufacturer’s instructions. WGS libraries were generated using standard protocols (Illumina). Libraries were sequenced to 15–30x genome coverage (2x150bp) on an Illumina NovaSeq 6000 system at the Hartwig Medical Foundation (Amsterdam, the Netherlands).

#### WGS read alignment and variant calling

WGS reads were mapped against the human reference genome (GRCh38) using the Burrows-Wheeler Aligner^43^ (v0.7.17) mapping tool with settings ‘bwa mem –c 100 –M’. Sequence reads were marked for duplicates using Sambamba^46^ (v0.6.8). Realignment was performed using the Genome Analysis Toolkit (GATK) (v4.1.3.0).^44^ A description of the complete data analysis pipeline is available at https://github.com/ToolsVanBox/NF-IAP (v1.3.0). Raw variants were called in multi-sample mode by using the GATK HaplotypeCaller and GATK-Queue with default settings and additional option ‘EMIT_ALL_CONFIDENT_SITES’. The quality of variant and reference positions was evaluated by using GATK VariantFiltration with options: “--filter-expression 'QD < 2.0' --filter-expression 'MQ < 40.0' --filter-expression 'FS > 60.0' --filter-expression 'HaplotypeScore >13.0' --filter-expression 'MQRankSum < −12.5' --filter-expression 'ReadPosRankSum < −8.0' --filter-expression 'MQ0 ≥ 4 && ((MQ0/(1.0 ∗ DP)) > 0.1)' --filter-expression 'DP < 5' --filter-expression 'QUAL <30' --filter-expression 'QUAL ≥ 30.0 && QUAL <50.0' --filter-expression 'SOR >4.0' --filter-name 'SNP_LowQualityDepth' --filter-name 'SNP_MappingQuality' --filter-name 'SNP_StrandBias' --filter-name 'SNP_HaplotypeScoreHigh' --filter-name 'SNP_MQRankSumLow' --filter-name 'SNP_ReadPosRankSumLow' --filter-name 'SNP_HardToValidate' --filter-name 'SNP_LowCoverage' --filter-name 'SNP_VeryLowQual' --filter-name 'SNP_LowQual' --filter-name 'SNP_SOR' -cluster 3 -window 10”.

#### Processing PTA data from external sources

Single-cell PTA-based WGS data (sra files) from cord blood tissue^17^ were downloaded from the Sequence Read Archive (accession code SRP178894) and extracted into bam files using the prefetch and sam-dump tools of the sratoolkit (v2.9.2).^59^ Samtools^45^ view (v1.3) was then used with the “-bf 1” argument to select for the paired reads and Picard SamToFastq (v2.24.1) was used with the “RG_TAG = ID” and “OUTPUT_PER_RG = true” arguments to generate fastq files. Seqkit^54^ replace (v2.2.0) was used to add a sample id to each read name, because they only consisted of a single read number and a number indicating whether it is the first or second read in the pair. Read alignment and variant calling were then performed as described above.

#### PTATO nextflow implementation

PTATO was implemented in Nextflow^48^ (v21.10.6.5661). Submodules were containerized and automatically downloaded by a container engine, allowing for an easy installation. A Docker image is provided for installation. Singularity (v3.8.7–1.el7) was used for this manuscript, though Docker will also work with a small change to the config. A full PTATO pipeline run (including base substitution filtering, indel filtering and SV calling) required 100–200 CPU hours per sample sequenced to a mean genome coverage of ∼15X.

#### PTATO resources

Next to the sample specific inputs, several general resource files were also used to run PTATO, which are listed in PTATO’s “resources.config” file. To make PTATO easy to install and more reproducible, these resource files are included with downloads of PTATO. First, the fasta file and accompanying indexes of the hg38 version of the human reference genome were downloaded from GATK (https://gatk.broadinstitute.org/hc/en-us/articles/360035890811). The input files necessary for the COBALT, GRIDSS2, and GRIPSS tools were downloaded from the Hartwig Medical Foundation (https://nextcloud.hartwigmedicalfoundation.nl/s/LTiKTd8XxBqwaiC?path=%2FHMFTools-Resources).^34^^,^^35^^,^^60^ A text file containing the centromere locations was downloaded from the UCSC (https://genome.ucsc.edu/cgi-bin/hgTables?hgsid=1424951119_QTS0nx5NshNSyspI7KDoJbVh9tci&clade=mammal&org=Human&db=hg38&hgta_group=map&hgta_track=centromeres&hgta_table=0&hgta_regionType=genome&position=chrX%3A15%2C560%2C138-15%2C602%2C945&hgta_outputType=primaryTable&hgta_outFileName=).^61^ A text file with the genomic coordinates of cytobands was also downloaded from the UCSC (https://genome.ucsc.edu/cgi-bin/hgTables?hgsid=1424951119_QTS0nx5NshNSyspI7KDoJbVh9tci&clade=mammal&org=Human&db=hg38&hgta_group=map&hgta_track=cytoBand&hgta_table=0&hgta_regionType=genome&position=chrX%3A15%2C560%2C138-15%2C602%2C945&hgta_outputType=primaryTable&hgta_outFileName=). A bed file with the genomic coordinates of simple repeats was downloaded from the UCSC for hg19 (http://genome.ucsc.edu/cgi-bin/hgTables?db=hg19&hgta_group=rep&hgta_track=simpleRepeat&hgta_table=simpleRepeat). A bed file with the genomic coordinates of gene bodies was downloaded from Ensembl for hg19.^62^ A bed file with replication timing data was generated as described previously.^6^ Files for which hg19 versions were downloaded were converted to hg38 using UCSCs LiftOver tool.^61^ Shapeit maps for hg38 were included with Shapeit (v4.2.2).^49^ Shapeit reference haplotype vcf files were downloaded from the 1000 genomes project (http://ftp.1000genomes.ebi.ac.uk/vol1/ftp/data_collections/1000G_2504_high_coverage/working/20201028_3202_phased/).

#### WGS quality control

Aligment summary metrics were generated for each sample using the CollectAlignmentSummaryMetrics tool from GATK (v4.1.3.0), while WGS metric files were generated using GATKs CollectWGSMetrics tool. Both tools were run using standard parameters. Next, the output of both tools was merged between all the samples and between the tools using R (v4.1.2). Finally, the R ggplot2^51^ (v4.3.0) package was used to generate quality control figures which are combined in a single pdf.

#### Somatic base substitution and indel filtering

The PTATO pipeline uses a multi-sample VCF file from a single individual and single bam files for each sample (including at least one germline control sample) as input. Preferably, the control samples are analyzed by bulk WGS, as we noted that removal of germline variants can be insufficient when using PTA-based WGS samples as controls. The somatic variant filtering tool SMuRF (https://github.com/ToolsVanBox/SMuRF), which is included in the PTATO pipeline, was used to remove germline and low-quality variants by applying several filters as described previously.^6^ Briefly, candidate somatic variants were included if they passed the following filters: no evidence in a paired bulk WGS control sample from the same individual; passed by VariantFiltration with a GATK phred-scaled quality score (QUAL) ≥ 100; base coverage of at least 10 (samples with ∼30X genome coverage) or 5 (samples with ∼15X genome coverage) in the PTA and paired control sample; a mapping quality (MQ) score of >55; and absence of the variant in a panel of unmatched normal human genomes. Additionally, heterozygous and homozygous base substitutions with a GATK genotype score (GQ) lower than 99 or 10, respectively, were removed. Indels with a GQ score lower than 99 in both PTA or paired control sample were removed. Somatic base substitutions with a variant allele frequency of <0.2 (for samples sequenced at ∼15X genome coverage) or <0.3 (for samples sequenced at ∼30X coverage) were removed. Somatic indels were required to have a variant allele frequency of at least 0.25. *The R-package VariantAnnotation57 (v1*.*42*.*1) was used to import and export VCF files in R*.

*The specific WGS samples that were used as* paired bulk WGS control samples to remove germline variants are indicated in Tables S1 and S2. Briefly, for the AML and FA patients we used bulk MSCs as germline reference. For the AHH-1 cell lines, we used a non-clonal bulk sample of the parental cell line as germline reference. For the cord blood samples, we used a clonally expanded HSPC sample from the same donor to remove germline variants. For patient PMCFANC02, no specific germline control sample was available, because the MSCs did not expand in culture. For this patient, we removed germline variants by selecting only variants that were private for each of the three samples.

Variant calling and filtering by SCAN2 was performed using standard settings (including the signature-based rescue step) as described in the manual (https://github.com/parklab/SCAN2/wiki).^18^ The somatic mutation burden estimated by SCAN2 for each sample was obtained from the.log files. Mutations at chromosome 17 were excluded in comparisons between PTATO and SCAN2, because SCAN2 repeatedly crashed while calling mutations on this chromosome.

#### Allelic imbalance analysis

Before modeling allelic imbalances, variants on each chromosome were phased separately using SHAPEIT^49^ (v4.2.2), with the raw vcf file containing all variants as its input. Additionally, the “sequencing” argument was used, SHAPEIT maps for the relevant reference genome were supplied to the map argument and a vcf with reference haplotypes was supplied to the reference argument.

For each candidate somatic variant, first all phased germline variants within 200,000 bp are selected to model allelic imbalance. To ensure only heterozygous germline variants are used, all variants that are not heterozygous in the bulk sample or do not have a dbSNP reference number were removed. After removing all germline variants that were not heterozygous in the sample, the allele depths of all variants phased to the second allele were swapped and the b-allele frequencies were calculated. Next, the b-allele frequencies were fitted with a locally weighted least-squares regression, which was used to predict the b-allele frequency of the candidate somatic variant. This regression was performed using the loess R-function with a degree of 2 and using the total allele depth of each variant as weights. Next, a binomial test was performed in R using both the predicted and observed b-allele frequency as well as the total allele depth of the candidate variant, to determine whether the observed allele frequency of the candidate variant matched the surrounding germline variants. The log of the p value from the allelic imbalance was then used for subsequent steps.

#### Selection of sequence context features

For each candidate somatic variant, the surrounding 10bp sequence context and mutation type were retrieved using functions modified from the MutationalPatterns R-package.^52^ The “closest” function from bedtools^47^ (v2.30.0) was used to identify the genes and simple repeat regions closest to the position of each candidate variant. Bedtools merge (with arguments "-d −1 -o min”) was used to ensure that each mutation is linked to only one feature of each feature list. To identify the transcriptional strand bias and replication timing for each somatic mutation, bedtools was used with the “intersect” argument. Some mutations were linked to multiple overlapping gene annotations. For the transcriptional stand bias this was solved by using bedtools with the “merge -d −1 -o distinct” arguments to check if a variant was present in the plus strand, minus strand or both. For the replication timing bedtools was used with the “merge -d −1 -o median” arguments to merge mutations that are present in multiple genes. Next, to merge the gene body, simple repeat, transcriptional strand bias, and replication timing features for each variant, bedtools was used with the “intersect” argument, after which the variants were merged using bedtools with the “merge -d −1 -o unique” arguments.

#### Linked read analysis using read-backed phasing

For each heterozygous candidate somatic variant, all sequencing reads overlapping the position of the variant were extracted from the sample’s bam file. Additionally, all heterozygous germline variants within the area spanned by the reads are extracted from the original input vcf. Next, for each germline variant each read that spans both the germline and somatic variant is checked. Each read that contains either the alternative alleles for both the germline and somatic variant or the reference alleles for both the germline and somatic variant is counted as a cis read. Other reads are counted as *trans* reads. If a candidate is real, then it would be expected that almost all reads are either cis or *trans*. Whether the variants are cis, *trans*, or mixed is then calculated based on a Bayesian likelihood score similar to the one used by SVTyper.^63^ The likelihood scores of the three options are then combined into a single Phred-scaled quality score. Candidate variants with a score of <100, between 100 and 1000 and >1000 were considered to be false positive, uncertain or true variants, respectively.

#### Random forest training

To obtain a set of true positive variants for training the RF model, base substitutions were selected that were detected in PTA samples of IBFM26, IBFM35, PB10268 and PMCAHH1-FANCCKO and also in bulk WGS-analyzed samples from the same individuals (Figure 1B, Tables S1, and S2). Somatic base substitutions with a linked read analysis score below 1 in these samples were included in the set of artifacts. Variants that were shared between PTA and bulk WGS samples and also had a linked read analysis score of less than 1 were excluded from both the true positive and the artifact datasets. Variants overlapping with copy number variants and regions of loss-of-heterozygosity in samples of IBFM26, IBFM35 and PMCAHH1-FANCCKO were excluded from training. Additionally, unique base substitutions detected in three umbilical cord blood HSPCs of donor PMCCB15 analyzed by PTA were considered artifacts, as the number of true mutations in the cord bloods is expected to be very low (20–50).^27^ Finally, the number of base substitutions in the artifact set was subsampled to be the same as the number of base substitutions in true positive set to result in a better class balance.

A random forest was trained on the previously described features with the randomForest (v 4.7–1) R package supplying the “mtry” argument with a value of 4. For some variants (<5%), no p value for the allelic imbalance or no replication timing value could be calculated (Figure S3D) and therefore they were excluded from the training. To be able to classify variants for which allelic imbalance or replication timing cannot be determined, two additional random forest models were trained: one without the allelic imbalance variable and one without both the allelic imbalance and the replication timing variables. The probability scores calculated by the three RF models were highly correlated (Figure S3E), showing that the additional RF models can effectively classify variants for which allelic imbalance or replication timing could not be determined.

The importance of each variable in the RF model was determined in two complementary ways. First, the mean decreases in Gini coefficient, which is a measure of the contribution of each feature to the homogeneity of the nodes and leaves in the random forest, were obtained from the standard output of model training by the randomForest R-package. Second, to test the impact of each feature on the performance of the model, performance was determined after consecutively removing the feature with the lowest mean decrease in Gini coefficient from the RF model. Each resulting RF model with decreasing numbers of features was applied to the training set to calculate the effect of removing the features one-by-one on the balanced accuracy (true positive rate plus true negative rate divided by 2) of base substitution classification (Figure S1B).

#### Candidate variant classification by PTATO

For each candidate somatic base substitution, PTATO’s main RF model was used to calculate a probability score to predict if a variant is a PTA artifact. A higher score indicates a higher probability that a variant is an artifact according to the RF. For less than 5% of the variants, the allelic imbalance or replication timing could not be determined (Figure S3D). For these variants, the probability scores of the second (without allelic imbalance) or third (without allelic imbalance and replication timing) RF model were used. Subsequently, two methods were used to determine a sample-specific cutoff value (variants above the cutoff were considered to be artifacts).

First, for each sample a group of likely true positive variants and a group of likely artifacts were selected by taking the variants with either a high (≥1000) or low (<1) linked read analysis score. These variants classified by the linked read analysis were used to validate the performance of the RF model. Precision and recall were calculated for a range of prediction score cutoff values (between 0 and 1 with increments of 0.01). The optimal linked read analysis cutoff was determined by taking the intersection of the precision-recall curves.

Second, a range of different cutoff values (from 0.1 to 0.8 with increments of 0.025) was taken and for each of these cutoffs the variants with a probability score below the cutoff were selected (leading to 29 groups of mutations). For all these 29 groups of mutations, a 96-trinucleotide mutation matrix was calculated using MutationalPatterns.^52^ Subsequently, the cosine similarities between all those groups were calculated using the calc_cosim_mutmat() function from MutationalPatterns. Hierarchical clustering of the cosine similarities was performed using the hclust() function in R (Euclidean distance with complete linkage) to generate two clusters: one cluster with low PTA probability cutoffs (and mostly true positives) and one cluster with relatively high cutoffs (and mostly false positives). The highest cutoff value in the cluster with true positives was taken as the cosine similarity cutoff.

Finally, the linked read analyses cutoff and cosine similarity cutoff were merged into a final cutoff that was used to classify variants as true or false positive. This was done by taking the mean of both cutoffs, or by only selecting the cosine similarity cutoff if the highest precision-recall value of the linked read analysis cutoff was below 0.7 (for example in case there were too few variants classified by the linked read analysis).

#### Somatic indel filtering

Candidate somatic indels were filtered based on recurrency in 139 PTA-based single-cell WGS samples of 22 unrelated individuals. For each included individual, indels occurring in bulk WGS data of the same individual were removed. Subsequently, all remaining somatic indel calls (genomic position, REF and ALT fields from the VCF files) from the PTA-WGS samples with a VAF >0.15 were collected in a MongoDB database. Indels occurring in at least two different individuals were exported from the database to the PTATO indel exclusion VCF file, which also contains the sample and individual counts and frequencies for each indel. Candidate indels in test samples that overlap with indels present in the exclusion VCF file were removed using the findOverlaps function of the GenomicRanges R-package (v1.48). Additionally, insertions in 5bp+ homopolymers were removed. For this, MutationalPatterns was used to determine the indel type and sequence context around candidate indels.

#### Mutation burden and signature analysis

The mutational patterns and signature analyses were performed using MutationalPatterns (v3.6.0).^52^ Mutational signatures were used from COSMIC (v3.2) as well as the previously described HSPC, PTA, and ENU signatures.^18^^,^^26^^,^^27^^,^^64^ The fit_to_signatures_bootstrapped function of MutationalPatterns (with parameters n_boots = 100 and method = ”strict”) was used to perform strict mutational signature refitting. Figures were made using ggplot2 (v3.4.1).^51^

CallableLoci from GATK v3.8.1 (with parameters --minBaseQuality 10 --minMappingQuality 10 --minDepth 8 --minDepthForLowMAPQ 10 --maxDepth 100) was used to determine the fraction of the sequenced genome that had sufficient coverage and quality for variant calling. Variants not overlapping with the callable regions determined by CallableLoci were excluded. Subsequently, all remaining variants on autosomal chromosomes were counted. To obtain the mutation burden, the mutation count was extrapolated by dividing it by the fraction of the genome that was surveyed (determined by CallableLoci), as previously described.^6^

A linear mixed-effects model was used to correlate the mutation burden in HSPCs from healthy donors and the age of the donors as previously described.^28^ This model was used to calculate the expected mutation burdens for the specific ages of the patients. The 95% confidence and 95% prediction intervals were calculated using the R package ggeffects (v1.1.0).^55^

#### *In silico* mixing of true and false variants

To determine how well PTATO can classify artifacts in datasets with different numbers of true base substitutions, PTATO was first applied to PTA samples PB15778-DX1BM-HSCPTAP1D12, PB32346-DX1BM-HSCPTAP3A7 and PMCCB15-CBCMP-PTAP3D10 to calculate the features of each base substitution. To obtain true positive variants, 800 base substitutions that were shared between the PTA samples (PB15778-DX1BM-HSCPTAP1D12 and PB32346-DX1BM-HSCPTAP3A7) and their corresponding bulk WGS samples (PB15778AMLBULK and PB32346-DX1BM-AMLBULK, respectively) were selected. From these 800 true positive variants, different numbers of variants (ranging from 100 to 800 with steps of 100) were randomly selected and merged with 465 base substitutions of PMCCB15-CBCMP-PTAP3D10 to create datasets with different ratios of true and false positives (with the values of the features from the samples in which the variants were originally detected). PTATO’s RF model was applied to each of these datasets to calculate how many true positive variants (variants that originated from samples of PB15778-DX1BM-HSCPTAP1D12 and PB32346-DX1BM-HSCPTAP3A7) remained after filtering and how many artifacts variants that originated from PMCCB15-CBCMP-PTAP3D10) were removed.

To test how well PTATO can classify variants with different mutational backgrounds, the 800 base substitutions shared between the PTA and bulk WGS samples from donors PB15778 and PB32346 were selected. For each mutational signature in the Cosmic Mutational Signatures database v3.2 (https://cancer.sanger.ac.uk/signatures/sbs/), the mutation type and the base up- and downstream features were modified in the feature tables of these 800 selected true base substitutions (while keeping all the other features the same). Subsequently, each set of the 800 true base substitutions with modified mutation spectra was merged with 465 base substitutions from PMCCB15-CBCMP-PTAP3D10. PTATO’s RF model was applied to each of these datasets to determine how many true positive variants with modified mutation spectra (variants that originated from samples of PB15778-DX1BM-HSCPTAP1D12 and PB32346-DX1BM-HSCPTAP3A7) remained after filtering and how many artifacts variants that originated from PMCCB15-CBCMP-PTAP3D10) were removed.

#### Normalization of copy number ratios for SV detection

GC-normalized read depth per 1000 basepair genomic window was calculated by COBALT (v1.11)^35^ (Figures S10C and S10D). Cosine similarities between raw genome-wide copy number profiles (1kb resolution) were calculated by using the cos_sim_matrix function of MutationalPatterns. A coverage panel-of-normals (PON) was generated by merging COBALT ratio files of 12 copy number neutral PTA samples. The total read counts from all windows of each sample were first normalized so that every sample has the same total amount of read counts. Subsequently, the mean readcount per bin over all normal samples in the PON was calculated. PTATO uses the coverage PON file to smoothen PTA-specific coverage fluctuations. First, the total read depth in a test sample is normalized to the same total amount of read counts in the coverage PON. Subsequently, the read counts in each window are divided by the mean read counts in the same window in the PON (Figure S10E). Additionally, the bottom and top 1% outlier windows in the PON file and the windows located within 1Mb distance of centromeres and telomers are excluded from the analysis.

The smoothened read counts were subsequently binned in 100kb windows (Figure S10F). The copynumber (v1.34.0) R-package with parameter “gamma = 100” was used to segment the median read count data in both the 100kb and 1kb windows^65^ (Figure S10G). The segments based on the 100kb resolution were used as raw copy number segments. The start and end coordinates of these raw copy number segments were fine mapped by taking the start and end coordinates of overlapping 1kb window-based segments.

To determine if the read count distribution within a segment was different from normal diploid segments in a sample, the read counts per 1kb from the top 25% of the segments with a mean copy number closest to 2 in the sample were selected (Figure S10H). For each segment, a *Z* score was determined by first subtracting the mean copy number in the segment by the mean copy number in the 25% segments with a copy number closest to 2, followed by dividing this number by the standard deviation of the copy number in the normal segments. The pnorm function in R was used to determine the significance in difference in coverage distributions between the segment and the 25% segments with a copy number closest to 2, which was called the “sample p value”. One-sided tests were used to determine if the copy number in the segment is either higher or lower than the diploid segments.

Each segment was overlapped with the mean read counts per 1kb bin in the coverage PON to compare the coverage distribution between the sample and the PON in the segmented region (Figure S10H). For each segment, a *Z* score was determined by first subtracting the mean copy number in the segment by the mean copy number in the PON, followed by dividing this number by the standard deviation of the copy number in the segment in the PON. The pnorm function in R was used to determine the significance in difference in coverage distributions in the segment between the test sample and the PON, which was called the “PON p value”. One-sided tests were used to determine if the copy number in the segment is either higher or lower than in the PON.

The segments with a sample p value <0.05 and a PON p value <0.2 were considered as potential copy number gains or losses in the later filtering steps that integrate the coverage and B-allele frequency segments.

#### Deviation of allele frequency calculations

VAFs of germline variants can be noisy in PTA-based WGS data due to uneven genome amplification, which impedes accurate copy number variant detection based on raw B-allele frequencies. To reduce noise due to uneven amplification, the VAFs of germline base substitutions were first binned in 100kb windows instead of taking separate B-allele frequencies of each individual variant. To determine a mean allele frequency for multiple variants in a bin, the deviation of allele frequency (DAF) was calculated by taking the absolute value after subtracting the VAF of each variant from 0.5 (which is the expected VAF for a perfectly amplified and sequenced germline variant). Thus, each variant has a DAF between 0 (corresponding to a VAF of 0.5) and 0.5 (corresponding to a VAF of 0 or 1). Subsequently, all DAF values of germline base substitutions are binned in 100kb genomic regions and the mean DAF for each region is calculated (Figure S11A). The copynumber R-package with parameter “gamma = 100” was used to segment the 100kb bins in crude DAF regions (Figure S11A). These crude segments were fine mapped by adjusting the start and end coordinates of the segments to the positions of the nearest germline SNVs (within 200kb distance of the segment) with similar DAFs as the segment.

Binning and segmenting were performed partly different from detect segments of potential copy number gains (Figure S11A). A small portion of genomic loci displayed loss-of-heterozygosity (LOH) because one of the alleles was not properly amplified by PTA. These artificial LOH regions may especially affect detection of copy number gains, because these regions have a relatively high DAF. Therefore, in parallel to binning and segmenting DAFs for detection of cnLOH and copy number losses as described above, PTATO also performed binning and segmenting after exclusion of all germline variants with a DAF >0.45 (corresponding to LOH) for detection of copy number gains (Figures S11A and S11B). Thus, PTATO determined two types of segments: one group of segments based on all germline variants for detection of copy number losses and cnLOH regions, and one group of segments based on only germline variants not displaying LOH for detection of copy number gains.

Finally, also the distribution of the VAFs of each germline variant was taken into account for CNV detection. The VAFs of germline variants in a normal diploid segment have a unimodal normal distribution around VAF = 0.5 (Figure S11B). In contrast, VAFs of germline variants in segments with copy number losses or gains are expected to have a bimodal distribution with modes at 0 and 1 for copy number losses and modes at 0.33 and 0.66 for copy number gains (with a copy number of 3) (Figure S11B). Therefore, PTATO used the Modes() function from the LaplacesDemon R-package (v16.1.6) to calculate the modes of the VAF distributions in each segment.

The segments with a DAF of more than 0.45 (corresponding to VAF <0.1 or >0.9) were considered to be LOH regions in the following integration of copy number segments and DAF segments (Figure S11B). The segments calculated after exclusion of LOH variants were used to select potential copy number gains. From these segments, only the segments that had 1) a mean DAF more than the average DAF in the sample and 2) more than one VAF distribution mode, of which one should be around 0.33 (+/− 0.12) and one should be around 0.66 (+/− 0.12), were selected as potential copy number gains (Figure S11B).

#### SV breakend calling and filtering

Somatic SV breakends were called by GRIDSS v2.13.2 and prefiltered by GRIPSS v1.9 using a corresponding bulk-sequenced germline control.^34^ StructuralVariantAnnotation v1.12.0 was used to import and export SV vcf files in R. The GRIPSS-filtered somatic breakends of 15 PTA-based samples of four unrelated individuals were merged using bedtools^47^ merge (v2.30.0). Breakend positions occurring within 2000bp of each other in multiple of these individuals were included in a breakend PON. Candidate breakends in other samples overlapping with the regions in the breakend PON were removed. Subsequently the normalized coverage and DAF of the SV candidates was calculated. Breakends of duplications were filtered if the DAF was less than 0.18 and/or the copy number ratio was <2.5. Breakends of deletions were filtered if the DAF was less than 0.4 and/or the copy number ratio was >1.5. Breakends with a coverage of more than 100 were also excluded for samples with a targeted genome coverage of 15x as many artifacts occur in these regions with excess coverage. Inversions were filtered if they only have one breakpoint junction instead of two. Additionally, all inversions less than 1kb in size were removed. Inter-chromosomal events were also filtered if they only have one breakpoint junction (instead of two), unless they were situated less than 100kb from a copy number variant. This exception rescues unbalanced translocations.

The GRIDSS-PURPLE-LINX pipeline (v1.3.2) developed by the Hartwig Medical Foundation^35^ was used for SV calling and filtering in bulk WGS samples.

#### Integration of coverage, allele frequencies and structural variant breakends

The coverage segments, DAF segments, and breakends of SV candidates were intersected to create the final list of filtered structural variants. Copy number variants were required to have both coverage and DAF support (based on the thresholds described above), but not necessarily breakend support, as many CNVs have start and/or end positions within repeat regions that are difficult to capture with PTA and/or short-read sequencing. Segments with a mean DAF of >0.45 (corresponding to VAFs of <0.1 and >0.9) that did not overlap with coverage segments of copy number losses or gains were considered to be copy number neutral loss-of-heterozygosity (cnLOH) regions. ggplot2^51^ and Circos^50^ (v0.69–9) were used for to visualize structural variants and karyograms. The SVs that were left after filtering were manually inspected by visualizing the reads in the bam files using the Integrative Genomics Viewer (IGV)^57^ for further validation.

### Quantification and statistical analysis

Statistical tests were performed with R and the rstatix and ggpubr R-packages. Details of each test are described in figure legends.

## Data Availability

• Raw whole genome sequencing data (BAM files) derived from human samples have been deposited at the European Genome-Phenome Archive (EGA) under accession number EGAS00001007288. They are available upon request if access is granted. Details on how to request access are available in the EGA repository. Additionally, de-identified somatic mutation data have been deposited at Mendeley Data (https://doi.org/10.17632/c3r9chw9rb.1) and are publicly available as of the date of publication. Original western blot images have also been deposited at Mendeley Data and are publicly available as of the date of publication. The accession numbers are listed in the key resources table. • All original code has been deposited at Github and is publicly available as of the date of publication. PTATO is freely available as open-source software (https://github.com/ToolsVanBox/PTATO, https://doi.org/10.5281/zenodo.8098608). Code used to analyze the data and create the figures is available at Github (https://github.com/ProjectsVanBox/PTATO, https://doi.org/10.5281/zenodo.8186323). • Any additional information required to reanalyze the data reported in this paper is available from the lead contact upon request.

## Supplemental information

Document S1. Figures S1–S12

Table S1. Study participant and whole-genome sequencing sample metadata, related to STAR Methods

Table S2. Metadata of whole-genome-sequenced cell lines, related to Figures 1 and 2

Table S3. Curated structural variant call set, related to Figure 5

Document S2. Article plus supplemental information
