# Supplementary material for: Comprehensive single-cell genome analysis at nucleotide resolution using the PTA Analysis Toolbox
Source: Cell Genom. 2023 Aug 23;3(9):100389. doi: 10.1016/j.xgen.2023.100389 (PMC10504672; doi:10.1016/j.xgen.2023.100389)
Supplement: Document S1. Figures S1–S12 [file mmc1.pdf]

Cell Genomics, Volume *3*

Supplemental information

Comprehensive single-cell genome analysis at nucleotide resolution using the PTA Analysis Toolbox

Sjors Middelkamp, Freek Manders, Flavia Peci, Markus J. van Roosmalen, Diego Montiel González, Eline J.M. Bertrums, Inge van der Werf, Lucca L.M. Derks, Niels M. Groenen, Mark Verheul, Laurianne Trabut, Cayetano Pleguezuelos-Manzano, Arianne M. Brandsma, Evangelia Antoniou, Dirk Reinhardt, Marc Bierings, Mirjam E. Belderbos, and Ruben van Boxtel

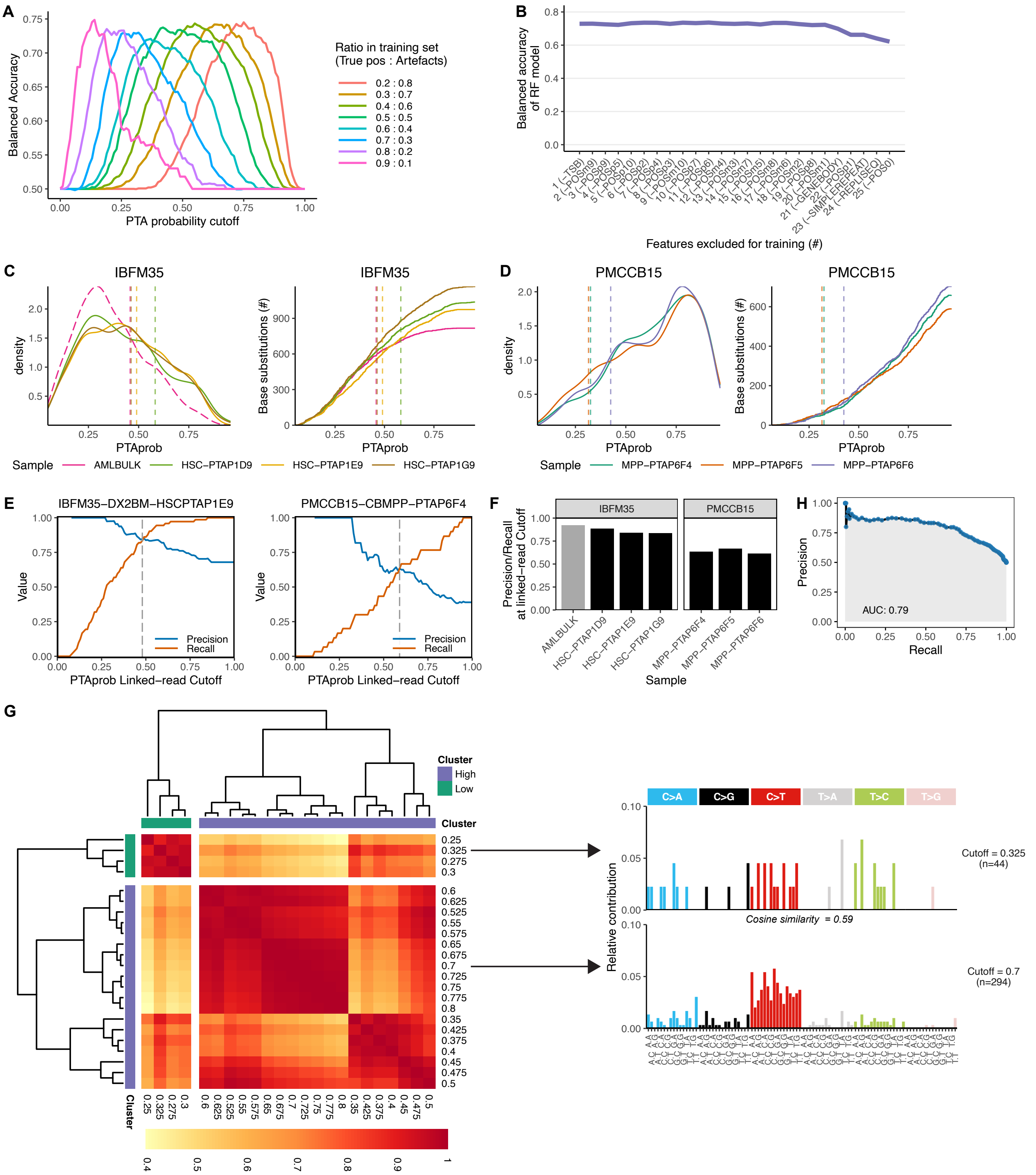

Figure S1. Calculations of optimal PTA probability cutoffs by PTATO, Related to Figure 1. (A) Training of the RF model was tested on different ratios of true and false positives to determine the optimal mix of variants. Changing these ratios mainly leads to a shift in PTA probability scores, while the optimal balanced accuracy remains the same (but achieved at a different cutoff value). The model trained at a 1:1 ratio shows the broadest range of cutoffs scores at which an optimal balanced accuracy is achieved, showing that this model is the most robust. **(B)** The effect on accuracy of cumulatively removing features one-by-one for training of the RF model. **(C)** Distributions (left) and cumulative distributions (right) of the PTA probability scores (PTAprob) of candidate base substitutions before PTATO filtering in one bulk WGS (with relatively low PTAprob scores) and three PTA-based WGS samples. Vertical lines indicate the sample-specific PTAprob cutoffs determined by PTATO. **(D)** Same as (C), but then for umbilical cord blood samples with low mutation burdens. **(E)** Precision and recall at different PTAprob cutoffs of a subset of base substitutions that could be classified as true or false positive by the linked read analysis. The linked read cutoff is determined by taking the PTA probability at minimal difference between the precision and recall. **(F)** Overview of the linked read precision-recall rates of samples in the training set. Samples with low mutations burdens can have low precision-recall rates, as shown here for cord blood donor PMCCB15, which requires an alternative method to calculate an optimal cutoff. **(G)** Heatmap showing the cosine similarities between 96-trinucleotide mutational profiles calculated for different PTAprob cutoffs in sample PMCCB15-CBMPP-PTAP6F4. Hierarchical clustering is used to make one cluster with low PTAprob cutoffs (containing most true positives) and one cluster with high PTAprob cutoffs (containing most artefacts). The highest value in the cluster with true positives is used as the cosine similarity cutoff (0.325 in this case). Two example profiles of the mutation sets at different cutoffs are shown on the right. **(H)** Precision-recall curve showing the performance of the random forest using all input variables on the out-of-bag training data for different probability cutoffs.

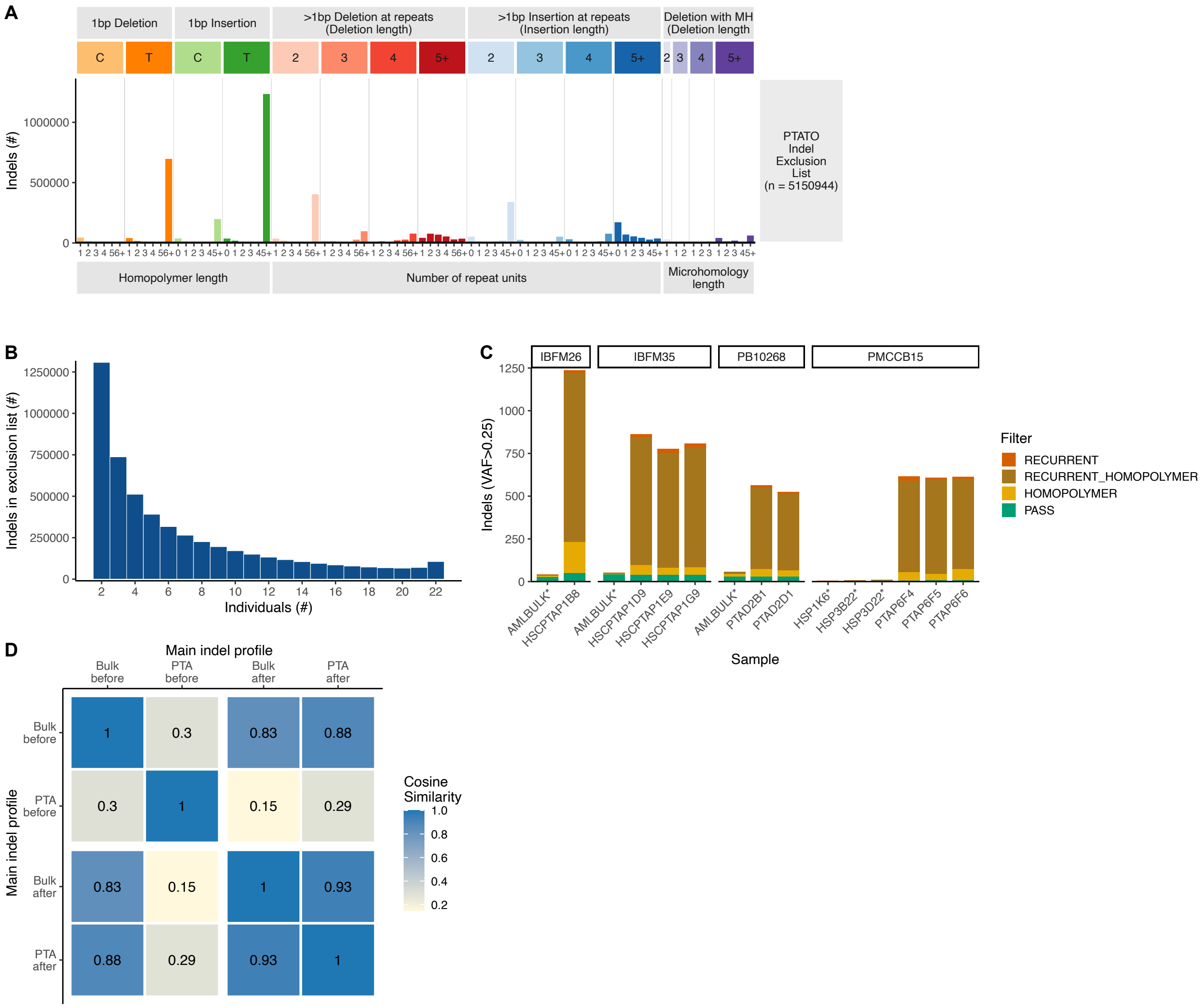

Figure S2. Indel filtering by PTATO based on recurrency and sequence context, Related to Figure 1. (A) Profile of the indels present in the list of recurrent indels that is used by PTATO to filter indel artefacts. The exclusion list contains mostly insertions at long homopolymers, but also recurrent deletions at long homopolymers. This indicates that just excluding insertions at long homopolymers is not sufficient to remove all indel artefacts. **(B)** Histogram showing in how many individuals (out of 22) the indels in the exclusion list are found. **(C)** PTATO filters indel artefacts by filtering insertions at long homopolymers (HOMOPOLYMER) and by filtering indels recurrent in multiple unrelated individuals (RECURRENT). This filtering removes most excess indels (the remaining indels are labelled with PASS), but also limits sensitivity to detect insertions in long homopolymer tracts. Samples indicated with an asterix (*) are bulk (non-PTA) WGS samples. **(D)** Heatmap showing the cosine similarities between the main indel spectra of the PTA- and bulk-WGS samples (from Figure 1H) before and after filtering by PTATO. The main indel spectra (16 channels) of the 6 PTA- and 3 bulk WGS samples of the AML patients shown in Figure 1H were merged by type (PTA, bulk, before and after filtering) before calculating the cosine similarities.

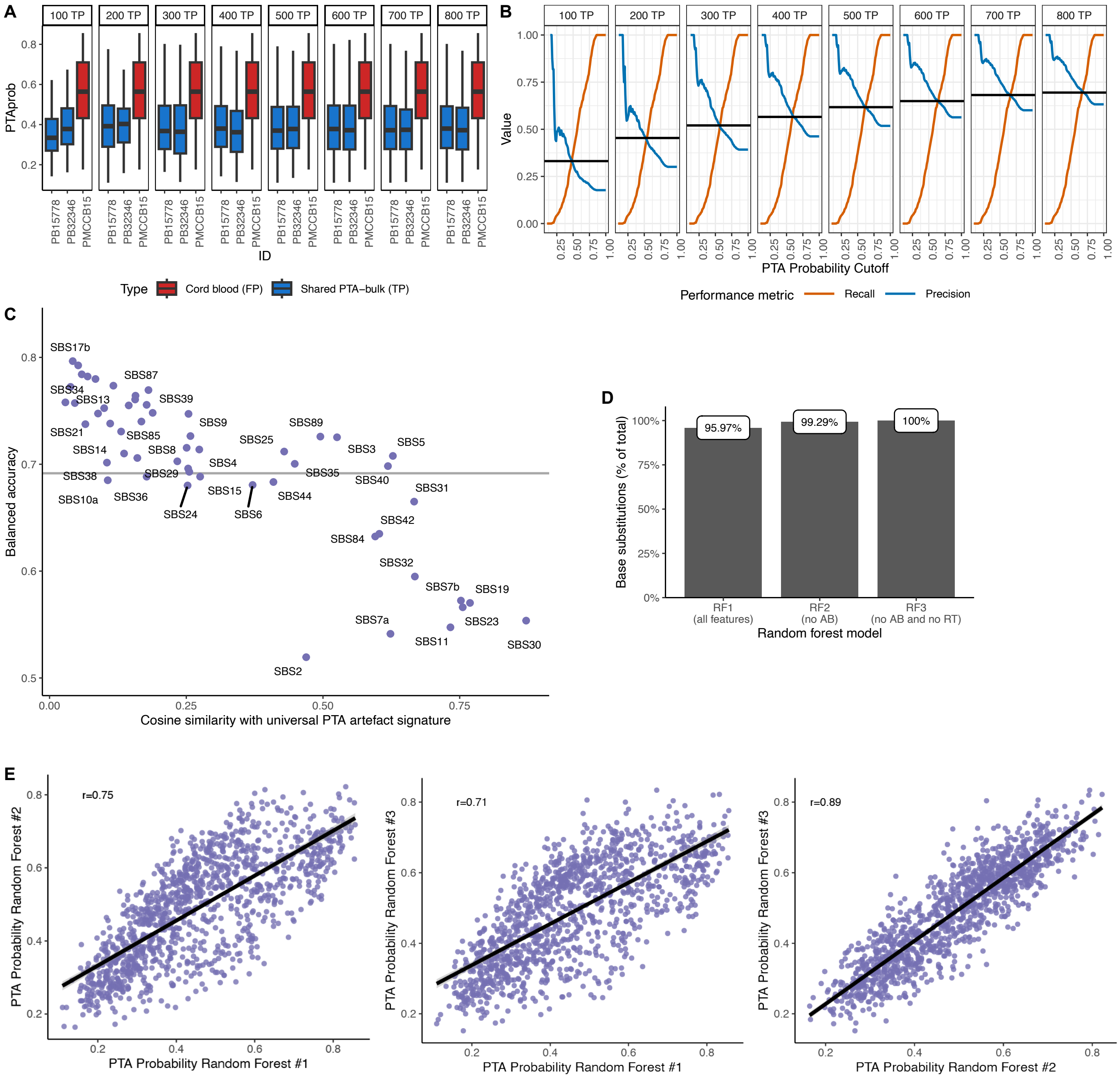

Figure S3. Validating PTATO performance by *in silico* mixing and mutating base substitutions, Related to STAR Methods. (A) PTA probability scores calculated by PTATO for base substitutions found in PTA-based WGS samples of two AML patients (PB15778 and PB32346) and one umbilical cord blood sample. For the two AML patients, only likely true positive (TP) base substitutions were included that were shared by the PTA sample and the bulk WGS sample. Most of these shared base substitutions have lower PTA probability scores compared to the base substitutions detected in the cord blood sample (most of which are PTA artefacts). **(B)** Precision-recall curves showing the performance of identification base substitution classification when mixing different amounts of true and false positives. True base substitutions were obtained by selecting mutations shared between PTA and bulk WGS samples of two AML patients. Different numbers of true positives (shown in the headers) were mixed with 465 base substitutions of a cord blood sample, which are considered artefacts. We note that roughly 10% of the base substitutions in the cord blood samples (~50 out of 465) are estimated to be real base substitutions, leading to an underestimation of the performance. **(C)** Balanced accuracy of PTATO in distinguishing *in silico* mutated true positive base substitutions from PTA artefacts (465 base substitutions from a cord blood sample). The trinucleotide contexts of sets of 800 base substitutions shared between bulk and PTA-based WGS samples from two AML patients were *in silico* mutated (while keeping the other RF features the same) to match the profiles of the depicted COSMIC mutational signatures. **(D)** For the majority of the base substitutions analyzed here (n=1265, 800 from the AML samples and 465 from the cord blood sample), all RF features could be determined. For some variants, values for the allelic imbalance (AB) and/or replication timing (RT) variables could not be calculated (for example due to low amplification quality or sequencing depth of the locus). For this small subset of variants, the PTA probabilities of the second or third random forest model (which exclude allelic imbalance and allelic imbalance plus replication timing, respectively) are used to determine if a variant is a PTA artefact. **(E)** Correlations (Pearson) between the PTA probability scores calculated by the first (all features), second (without the allelic imbalance feature) and third (without the allelic imbalance and replication timing features) random forest models for the base substitutions (dots) analyzed here. As only a small number of variants cannot be analyzed by random forest 1 and because the probabilities calculated by the three models are highly correlated, random forest 2 and 3 are only expected to have a minor effect on variant filtering. Nevertheless, they can be useful to rescue the small subset of variants that cannot be analyzed with the primary model.

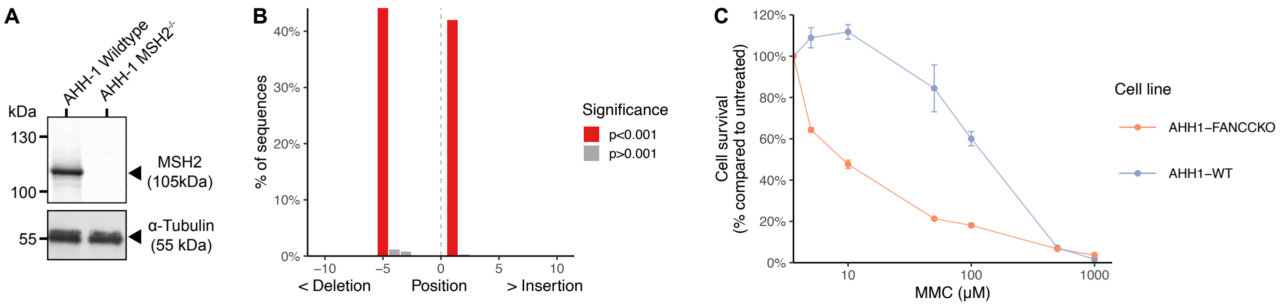

Figure S4. Validation of *MSH2* and *FANCC* knockout status in AHH-1 cell lines, Related to Figure 2. (A) Western blot showing the absence of MSH2 protein expression in the AHH-1 *MSH2*$^{-/-}$ clonal cell line. **(B)** TIDE analysis detects a 5-basepair deletion and 1-basepair insertion introduced by CRISPR/Cas9 in the *FANCC* gene of the AHH-1 *FANCC*$^{-/-}$ clonal cell line. Due to the absence of high quality antibodies, western blotting could not be performed to study FANCC protein expression. Therefore, we used PCR and Sanger sequencing followed by TIDE decomposition, in addition to a Mitomycin C (MMC) sensitivity assay, to confirm knockout status. The presence of the biallelic indels in *FANCC* was also confirmed in the WGS data (data not shown). **(C)** MMC sensitivity assay showing the hypersensitivity of the AHH1 *FANCC*$^{-/-}$ clonal cell line to the DNA cross-linking agent MMC. This finding provides additional support for the knockout status of *FANCC* in this cell line, as cells of patients with FA are known to display MMC hypersensitivity. Mean survival values from triplicate experiments are shown and error bars indicate standard deviations.

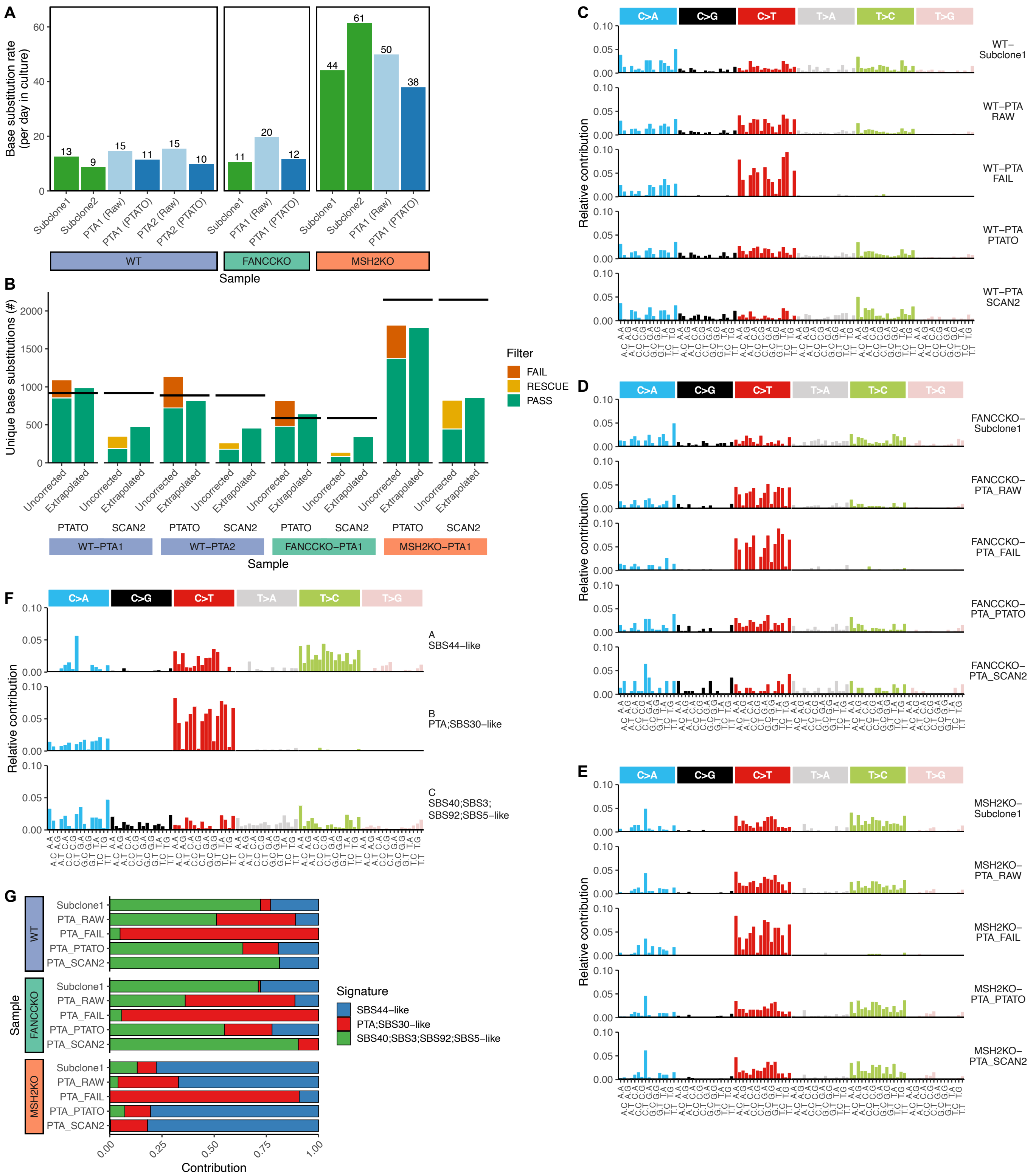

Figure S5. Single base substitution filtering in PTA-based WGS data of AHH-1 cell lines by PTATO, Related to Figure 2. (A) Number of base substitutions aqcuired per day in culture between single cell steps in subclones analyzed by bulk WGS (green) and PTA samples before (lightblue) and after (darkblue) PTATO filtering. **(B)** Number of unique base substitutions not present in the (sub)clones reported by PTATO and SCAN2 before and after extrapolation. PTATO detects more base substitutions, requiring less extrapolation to estimate the true base substitutions burden in a cell. The horizontal black lines indicate the expected number of base substitutions based on the days in culture since the previous single-cell step and the mutation rate in the corresponding subclones. **(C-E)** The 96-trinucleotide mutational profiles of the wildtype (WT) (C), FANCC-KO (D) and MSH2-KO (E) AHH-1 cells assessed by WGS after clonal expansion or after PTA. The variant calls before PTATO filtering (RAW) still contain numerous PTA artefacts. The profiles of the variants removed by PTATO are shown in the middle panels (PTA_FAIL). **(F)** 96-trinucleotide profiles of the base substitution signatures extracted by non-negative matrix factorization (NMF). One signature resembles the PTA artefact signature (red), one resembles the background signature for AHH-1 cells (green) and one resembles signatures found in mismatch repair deficient cells (blue). **(G)** Contribution of the signatures extracted by NMF (F) to the mutational profiles of each sample. The mutations removed by PTATO (PTA_FAIL) are mostly refitted to the PTA artefact signature. The mutational profiles of the PTA samples filtered by PTATO and SCAN2 are more similar to the profiles of the subclones analyzed by bulk WGS than the unfiltered (PTA_RAW) samples.

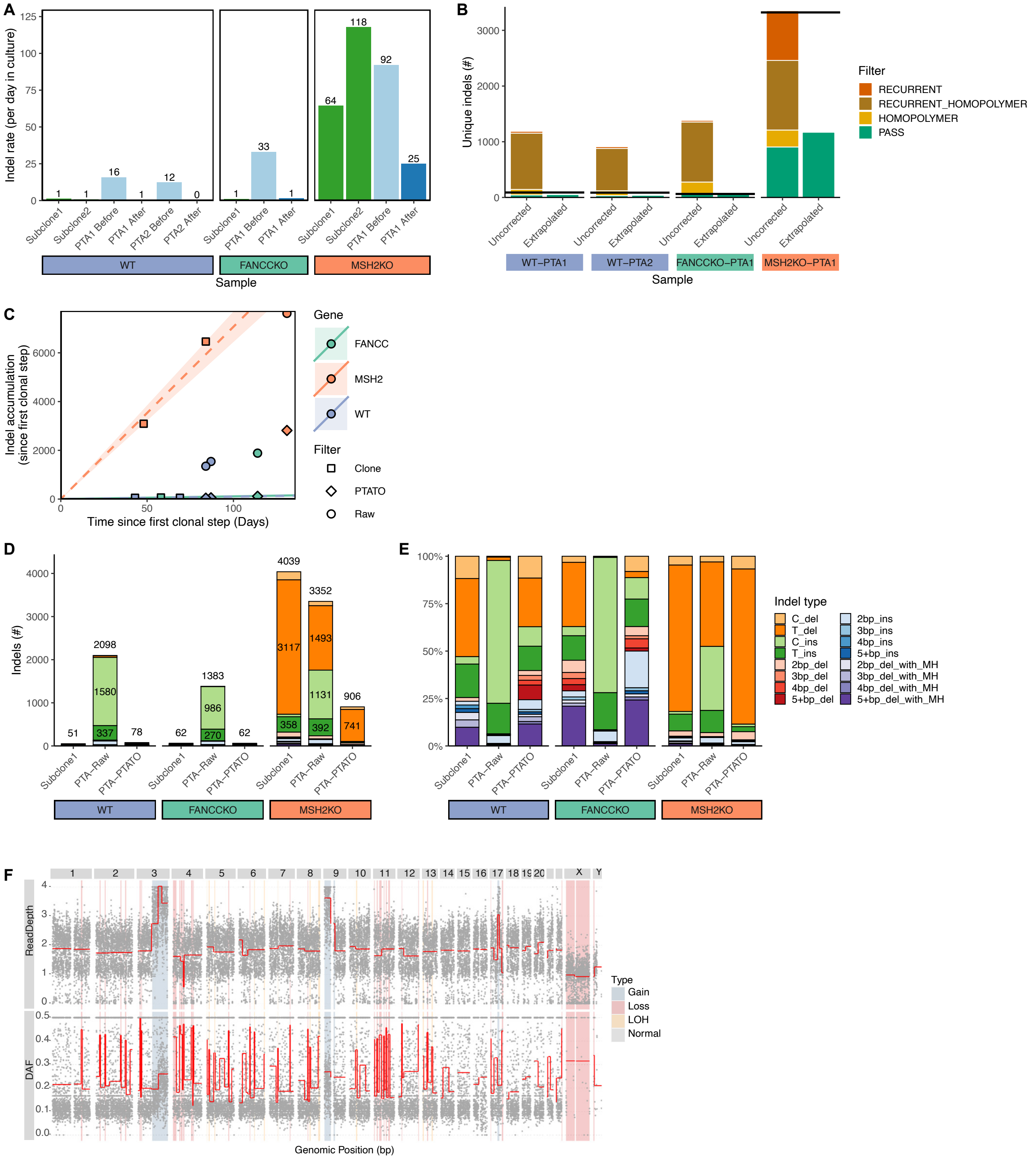

Figure S6. Indel filtering in PTA-based WGS data of AHH-1 cell lines by PTATO, Related to Figure 2. (A) Number of indels acquired per day in culture between single cell steps in subclones analyzed by bulk WGS (green) and PTA samples before (lightblue) and after (darkblue) PTATO filtering. **(B)** Number of unique indels not present in the (sub)clones reported by PTATO before and after extrapolation. The horizontal black lines indicate the expected number of indels based on the days in culture since the previous single-cell step and the indel accumulation rate in the corresponding subclones. **(C)** Accumulation of indels since the first clonal step. The circles and diamonds indicate the number of indels detected in the PTA samples before and after PTATO filtering, respectively. **(D)** Number of indels (not present in the preceding clonal step) in the subclones analyzed by bulk WGS and the PTA samples before (Raw) and after PTATO filtering. More than a thousand artificial indels are detected in the wildtype and FANCC$^{-/-}$ PTA samples. **(E)** Relative contributions of the different types of indels (not present in the preceding clonal step) detected in the subclones analyzed by bulk WGS and the PTA samples before (Raw) and after PTATO filtering. PTATO mostly removes 1-basepair (bp) insertions. **(F)** Copy number and deviation-of-allele frequency (DAF) plots of sample PMCAHH1-MSH2KO-C27E06SC51B06-PTAP1E7. This sample has many loss-of-heterozygosity (LOH) regions, indicating a lower quality genome amplification by PTA. MH, microhomology.

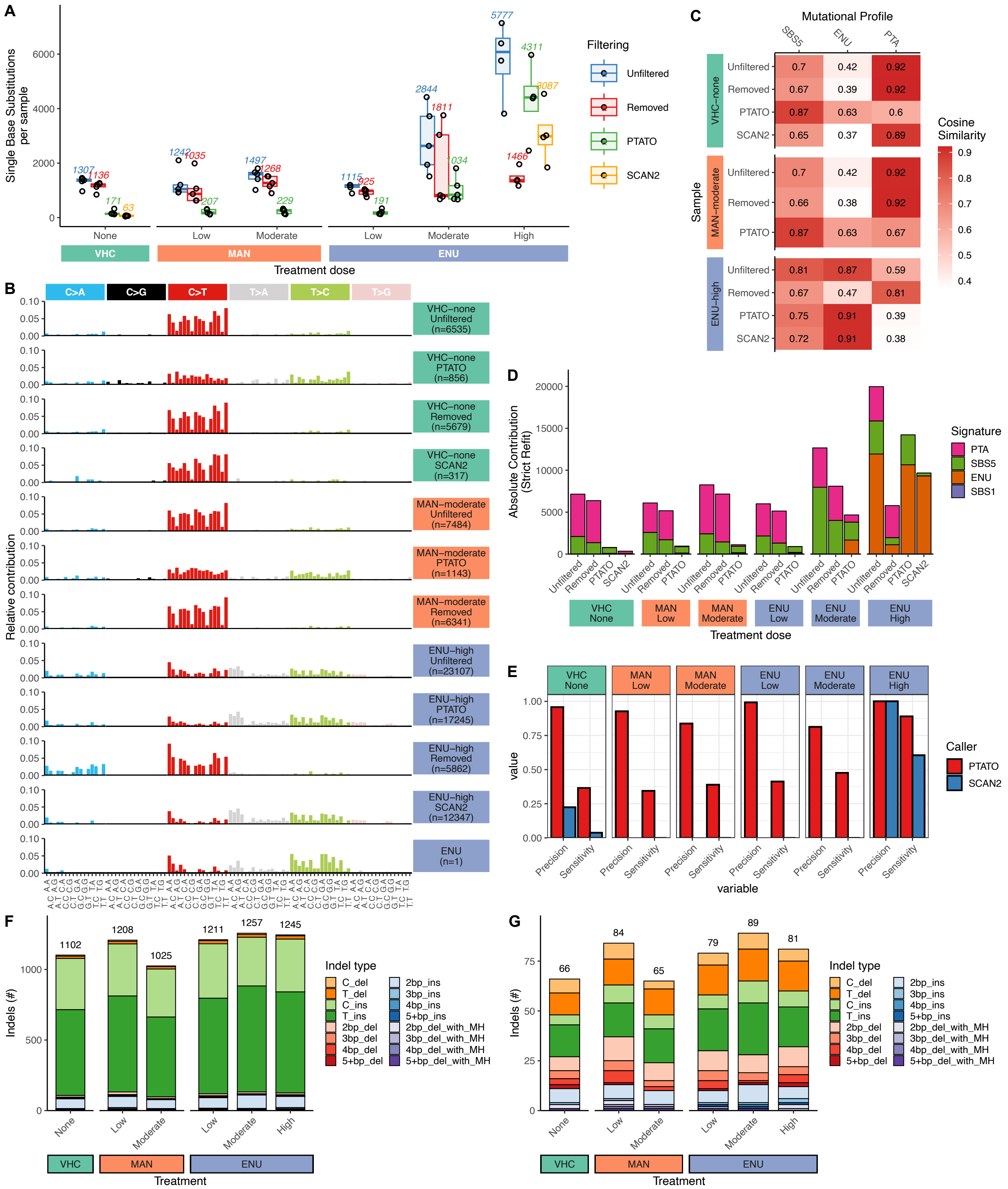

Figure S7. PTATO accurately filters PTA artefacts from a PTA-based WGS dataset of cord blood cells, Related to Figure 2. (A) Boxplot showing the number of base substitutions for human cord blood samples treated with different concentrations of a vehicle control (VHC; n = 5), D-mannitol (MAN; low: n = 5, moderate: n = 5) or N-ethyl-N-nitrosourea (ENU; low: n = 5, moderate: n = 5, high: n = 4). Numbers above the boxes indicate the mean base substitution burden per sample in each treatment group. **(B)** The 96-trinucleotide profiles of the base substitutions in the indicated treatment groups before ("Unfiltered") or after ("Filtered") PTATO filtering or the base substitutions removed by PTATO ("Removed") or the mutations detected by SCAN2. The bottom panel shows the profile of the mutational signature that has been previously associated with ENU-treatment. **(C)** Cosine similarities of the mutational profiles of the base substitutions that are present before ("Unfiltered") or after PTATO filtering ("PTATO"), or that are removed by PTATO or detected by SCAN2, with the SBS5-, ENU- and PTA mutational signatures. Variants detected by SCAN2 in the VHC samples show a strong similarity to the PTA artefact signature, suggesting it mostly detects artefacts in these samples. **(D)** Contributions of the PTA, SBS1, SBS5 and ENU mutational signatures to the profiles of the unfiltered, removed and filtered base substitutions determined by a bootstrapped strict mutational refit. PTATO mostly removes mutations associated with the PTA mutational signature, while keeping the mutations associated with SBS5 and the ENU mutational signatures. The base substitutions were pooled for each treatment dose. **(E)** Precision and sensitivity of base substitution detection by PTATO and SCAN2. Precision is defined as 1 minus the fraction of base substitutions refitted to the PTA artefact signature. Sensitivity is defined as mean contribution of SBS1, SBS5 and ENU-signatures in the Unfiltered call sets minus mean contribution of SBS1, SBS5 and ENU-signatures in the PTATO and SCAN2 call sets. **(F)** Mean numbers and types of indels found per sample in each treatment group before filtering by PTATO. **(G)** Mean numbers and types of indels found per sample in each treatment group after filtering by PTATO. PTATO removes over a thousand indels per sample, mainly C- and T-insertions at homopolymers. As has been shown before, treatment with ENU did not cause an increase in indel burden. MH, Microhomology.

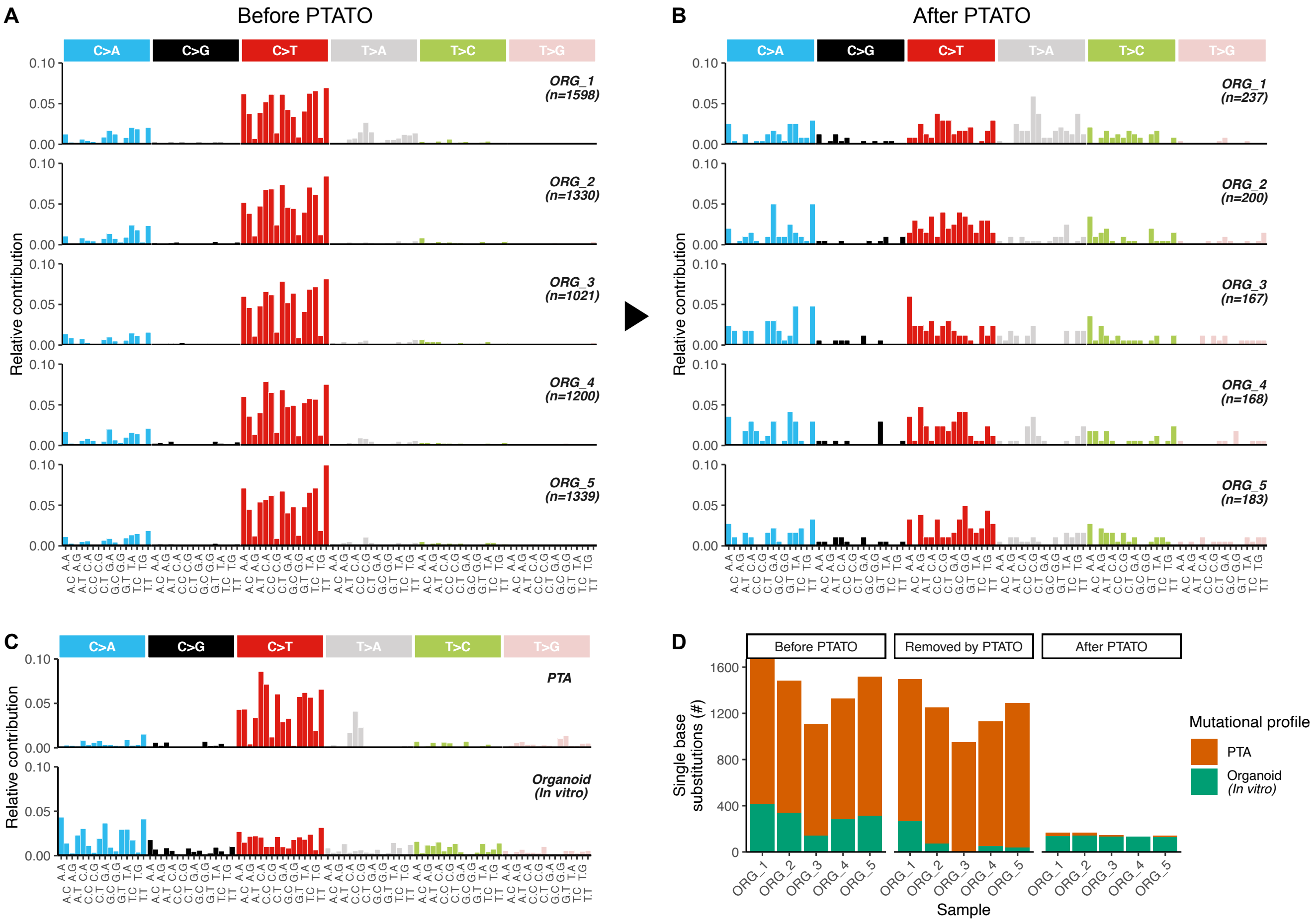

Figure S8. Filtering of PTA-based WGS data of single intestinal organoid cells by PTATO, Related to Figure 2. (A) The 96-trinucleotide mutational profiles of five single intestinal organoid cells analyzed by PTA-based WGS, before PTATO filtering. **(B)** The 96-trinucleotide mutational profiles of five single intestinal organoid cells analyzed by PTA-based WGS, after PTATO filtering. **(C)** The 96-trinucleotide mutational profiles of the PTA artefact (top) and organoid (bottom) mutational signatures used for signature refitting. The profile of base substitutions that accumulate during *in vitro* culture of intestinal organoids was previously determined by analysis of the subclonal mutations in WGS data of clonal organoids. **(D)** Contribution of mutational signatures to the base substitution profiles of the five organoid cells determined by bootstrapped signature refitting. Filtering by PTATO removes nearly all base substitutions that could be attributed to the PTA artefact signature, showing that it is also applicable to non-hematological PTA samples.

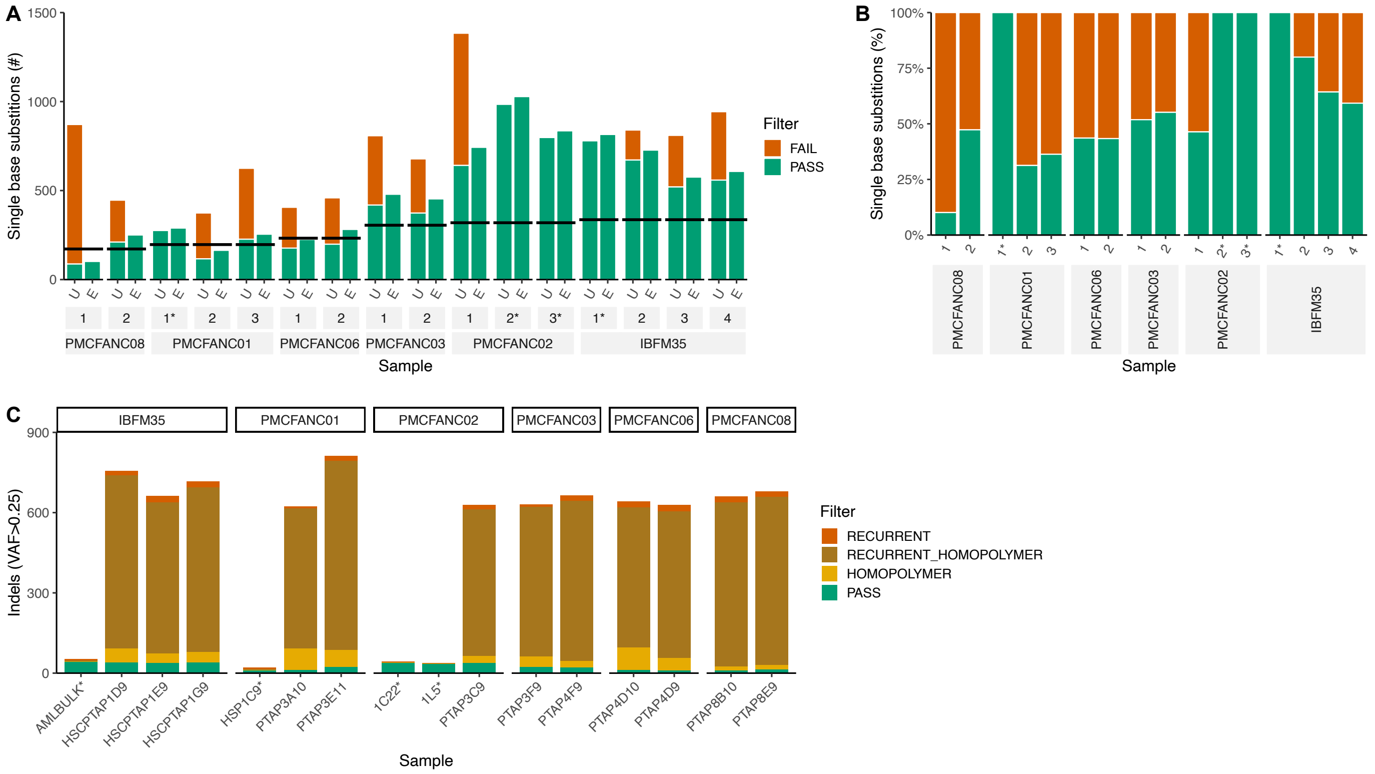

Figure S9. PTATO filtering of single base substitutions and indels in HSPCs of patients with FA, Related to Figure 3. (A) Absolute number of single base substitutions that passed (PASS) or failed (FAIL) filtering by PTATO, before (U = unfiltered) and after (E = extrapolated) extrapolation based on CallableLoci. The horizontal black lines indicate the expected number of base substitutions for each individual based on their age. Samples not amplified by PTA are marked with an asterisk. **(B)** Relative amount of single base substitutions that that passed or failed filtering by PTATO. **(C)** Number of indels per sample that passed filtering by PTATO or that were removed by PTATO because they are present in the recurrent indel filter list (RECURRENT) or are insertions in homopolymer regions (HOMOPOLYMER), or both (RECURRENT_HOMOPOLYMER).

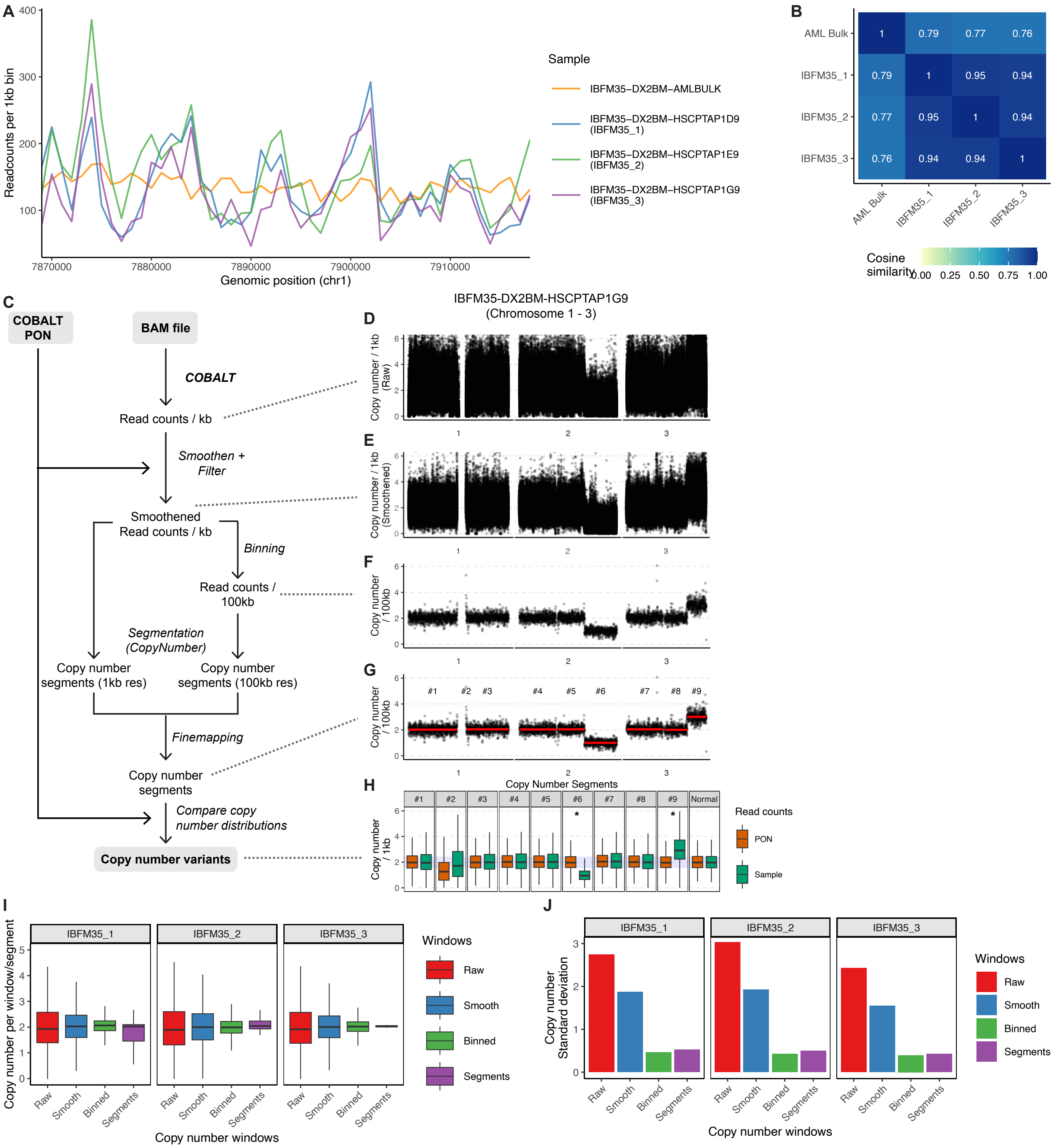

Figure S10. Copy number variant detection by PTATO based on read depth, Related to Figure 5. (A) Coverage profiles determined by COBALT (at 1kb resolution) of three PTA samples and one bulk WGS samples in a 50kb region on chromosome 1. **(B)** Heatmap showing the cosine similarities between the genome-wide coverage profiles (1kb resolution). **(C)** Overview of the first part of copy number filtering (based on coverage) performed by PTATO. **(D)** Example of a copy number profile (1kb resolution) of three chromosomes determined by COBALT, before any filtering by PTATO. **(E)** Copy number profile (1kb resolution) after smoothening by PTATO using the PON. **(F)** Copy number profile at 100kb resolution after binning the smoothened read counts by PTATO. **(G)** Copy number profile (100kb resolution) which shows the calculated copy number segments as red horizontal lines. The detected segments are labelled by the numbers above the plot (#1 to #9). **(H)** Distributions of the copy numbers (1kb resolution) in the 12 samples in the PON (containing normal diploid samples) and the test sample (IBFM35-DX2BM-HSCPTAP1G9) for each of the 9 detected segments (on the three chromosomes) with similar copy numbers. Additionally, in the last panel the coverage distributions in the top 25% of the bins closest to copy number 2 are shown to depict the variation in copy number in regions that are considered to be normal diploid. These coverage distributions were used by PTATO to determine which segments are potentially copy number gains or losses, as indicated by the asterisk. In later steps, these segments of copy number variant candidates were intersected with segments with divergent germline variant allele frequencies to generate the final copy number variant call set. **(I)** The effects of each consecutive coverage filtering step on the variance in copy number between genomic windows. **(J)** The standard deviation of the copy numbers in each genomic window after each coverage filtering step. This shows that each filtering step further reduces the variance in copy number profiles.

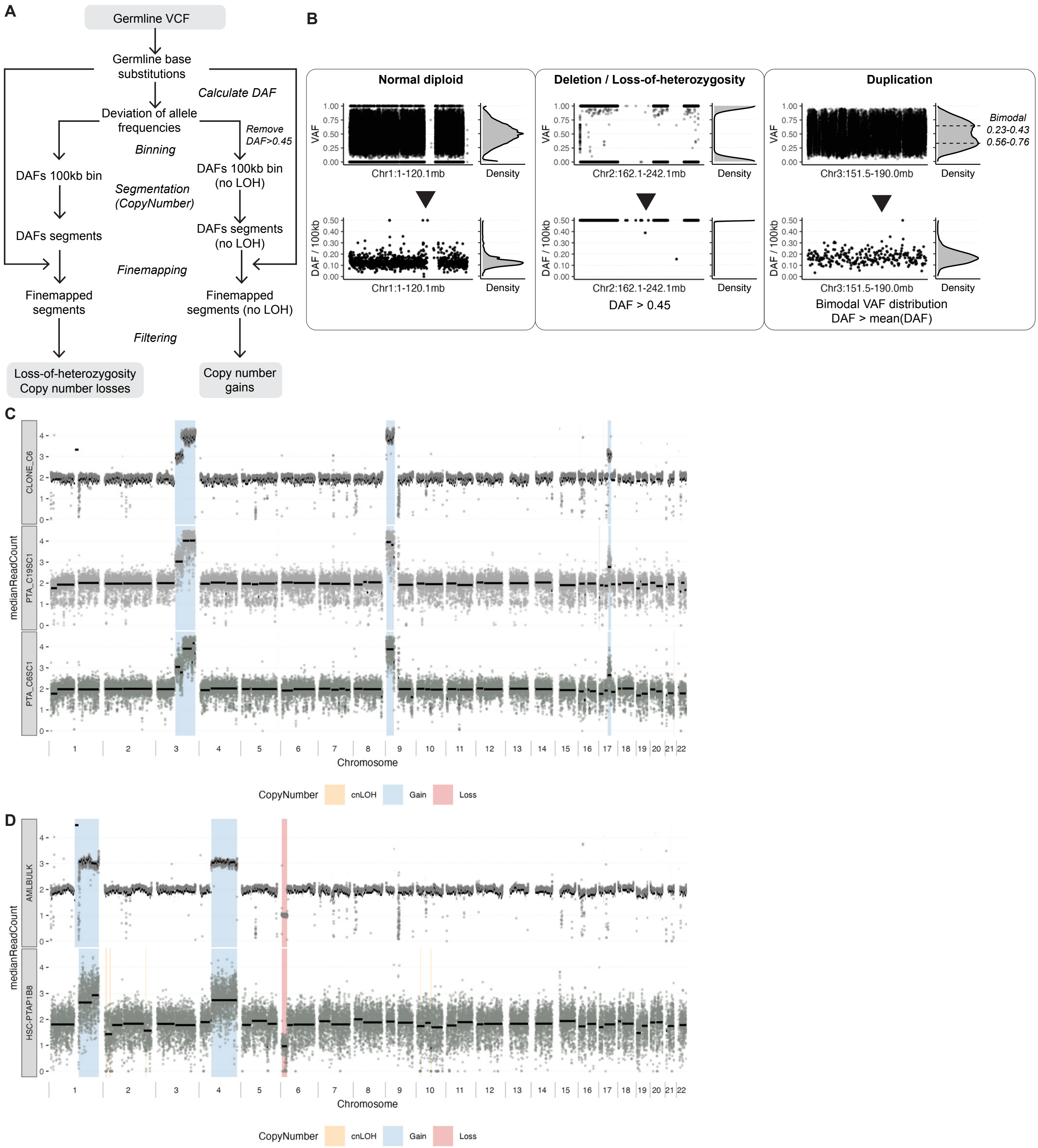

Figure S11. Copy number calling with allele frequencies germline base substitutions, Related to Figure 5. (A) Schematic overview of the filtering steps performed by PTATO to identify copy number changes based on allele frequencies of germline base substitutions. Filtering for loss-of-heterozygosity (LOH) and deletions on the one hand, and copy number gains on the other hand were performed in parallel. For detection of copy number gains, first all germline variants with a DAF of >0.45 (corresponding to a loss of heterozygosity) were removed. This was done to minimize the effects of LOH that was caused to uneven DNA amplification by PTA on detection of duplicated regions. **(B)** Examples of VAF and DAF distributions of a copy number neutral region (left), a genomic region with a copy number loss (center) and a genomic region with a copy number gain (right) in sample IBFM35-DX2BM-HSCPTAP1G9. These examples depict how PTATO made use of germline variant allele frequencies as a part to identify copy number variants. LOH and deletions events are called if the mean deviation of allele frequencies (DAF) in a segment was more than 0.45. Duplications were called if the mean DAF of a segment is higher than the mean DAF in the entire sample and if there was a bimodal distribution of the VAFs of germline variants in a segment with modes of ~0.33 and ~0.66. **(C)** Copy number profiles (at 100kb resolution) of one bulk WGS (CLONE_C6) and two single-cell PTA-based WGS samples of clonal AHH-1 cell lines. Colored background shadings show the copy number calls made by PTATO (for the PTA-based WGS samples) or PURPLE (for the bulk WGS sample). The black horizontal lines depict the copy number segments determined by PTATO (for the PTA-based WGS samples) or COBALT (for the bulk WGS sample). **(D)** Copy number profiles (at 100kb resolution) of one bulk WGS (AMLBULK) and one single-cell PTA-based WGS sample (HSC-PTAP1B8) of AML patient IBFM26. Colored background shadings show the copy number calls made by PTATO (for the PTA-based WGS sample) or PURPLE (for the bulk WGS sample). The black horizontal lines depict the copy number segments determined by PTATO (for the PTA-based WGS sample) or COBALT (for the bulk WGS sample).

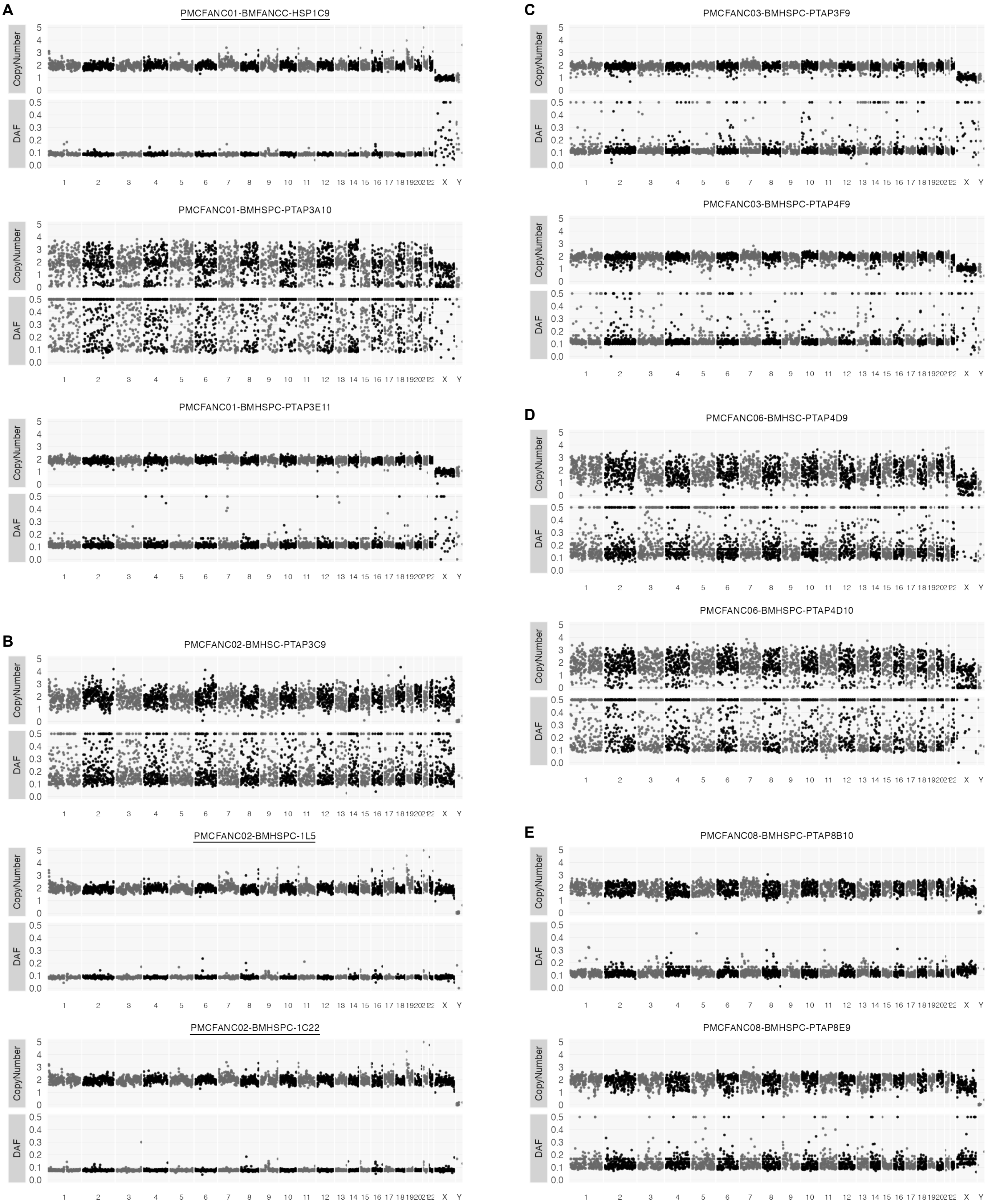

Figure S12. No large chromosomal rearrangements detected in the HSPCs of patients with FA, Related to Figure 5. (A-E) Copy number and DAF plots (at 1Mb resolution) after PTATO filtering of 12 analyzed HSPCs of 5 patients with FA. There is variability in the PTA quality between the single cells, leading to a lower sensitivity to detect SVs in some samples with relatively low quality (e.g. PMCFANC01-BMHSPC-PTAP3A10 and PMCFANC06-BMHSPC-PTAP4D10). Names of samples that were analyzed by WGS after clonal expansion (instead of PTA) are underlined.
